# Enterotoxigenic *Escherichia coli* heat labile enterotoxin affects neutrophil effector functions via cAMP/PKA/ERK signaling

**DOI:** 10.1080/19490976.2024.2399215

**Published:** 2024-09-16

**Authors:** Jinglin Ma, Leen Hermans, Matthias Dierick, Hans Van der Weken, Eric Cox, Bert Devriendt

**Affiliations:** Laboratory Immunology, Department of Translational Physiology, Infectiology and Public Health, Faculty of Veterinary Medicine, Ghent University, Merelbeke, Belgium

**Keywords:** Heat labile enterotoxin, ETEC, neutrophils, pig, cAMP/PKA/ERK signaling

## Abstract

Enterotoxigenic *Escherichia coli* (ETEC) are a major cause of diarrheal illness in humans and animals, induced by enterotoxins produced by these pathogens. Despite the crucial role of neutrophils in combatting bacterial infections, our understanding of how enterotoxins impact neutrophil function is limited. To address this knowledge gap, we used heat-labile enterotoxin (LT) and heat-stable enterotoxin a (STa) to investigate their impact on the effector functions of neutrophils. Our study reveals that pSTa does not exert any discernible effect on the function of neutrophils. In contrast, LT altered the migration and phagocytosis of neutrophils and induced the production of inflammatory factors via activation of cAMP/PKA and ERK1/2 signaling. LT also attenuated the release of neutrophil extracellular traps by neutrophils via the PKA signaling pathway. Our findings provide novel insights into the impact of LT on neutrophil function, shedding light on the underlying mechanisms that govern its immunoregulatory effects. This might help ETEC in subverting the immune system and establishing infection.

## Introduction

Enterotoxigenic *Escherichia coli* (ETEC) is a major cause of diarrhea in children in and travelers to ETEC endemic regions as well as in livestock species, including swine.^1–3^ ETEC strains have two main classes of virulence factors: colonization factors or fimbriae, and enterotoxins.^4,5^ While the fimbriae are critically important to establish an infection by mediating the adhesion of ETEC to the gut epithelium, the enterotoxins trigger diarrhea, facilitating nutrient acquisition or bacterial transmission. Two types of secreted enterotoxins can be distinguished, the heat-labile enterotoxin (LT) and the heat-stable enterotoxins (STs).^4,6^ LT is an AB_5_ toxin that binds to the gut epithelium via its pentameric B subunit in a ganglioside M1 (GM1)-dependent manner. Upon internalization, the A subunit activates adenylate cyclase, leading to increased intracellular cyclic adenosine monophosphate (cAMP) levels. This in turn activates protein kinase A (PKA), leading to the opening of the cystic fibrosis transmembrane regulatory channel (CFTR) and the inhibition of the Na+/H+ ion exchanger isotype 3 (NHE3). Together, this causes the efflux of electrolytes and water into the gut lumen.^7,8^ Heat-stable enterotoxin a (STa, a short 18–19 aa peptide) on the other hand acts by activating guanylate cyclase on intestinal epithelial cells, leading to increased cyclic GMP (cGMP) levels. This in turn affects the function of both ion channels, resulting in a disruption of the electrolyte balance, which ultimately leads to diarrhea.^9^

The enterotoxicity of LT and STs is well known, along with the pathways they trigger in gut epithelial cells which ultimately lead to diarrhea. Intestinal epithelial cells respond to ETEC and its virulence factors by producing inflammatory mediators which attract and inform innate immune cells and help in coordinating immune responses to clear ETEC infections.^10–12^ In addition to this indirect effect on immune cells, LT is a potent immunogen and activates dendritic cells to promote mucosal immune responses.^13,14^ Intriguingly, LT also decreases phagocytosis by macrophages and reduces inflammation by driving the polarization of alternatively activated macrophages.^15^ The impact of STs on immune cells is less studied, although recent data indicate that STa does not directly affect macrophage function.^6,15^ Whether LT and STa can affect the function of other innate immune cells, like neutrophils, is unknown.

Neutrophils have potent antimicrobial activities and play a critical role in protecting the host against bacterial pathogens, also at barrier sites. Neutrophils migrate in abundant numbers to injured or infected sites, where they perform effector functions to contain and eliminate pathogens. These effector functions include the phagocytosis of bacteria, the release of host defense proteins, the generation of reactive oxygen species (ROS) and the formation of neutrophil extracellular traps (NETs).^16^ These web-like chromatin structures are released by the neutrophils and are composed of DNA and various antimicrobial components to trap and kill bacteria.^17^ However, some pathogens have evolved mechanisms to evade killing by NETs and even use these to their own benefit.^17^ In addition, neutrophils release cytokines and chemokines to help in orchestrating and regulating immune responses to infections.^18^ Intriguingly, neutrophils can migrate across the gut epithelium into the intestinal lumen both in steady state and during inflammation as well as ETEC infection.^19–22^ This offers an opportunity for direct contact between neutrophils and ETEC enterotoxins in the gut lumen.

Given the essential role of neutrophils in the defense against bacterial infections and in coordinating immune responses, we aimed to investigate whether the ETEC-derived enterotoxins LT and pSTa (STa secreted by porcine ETEC strains) can affect the function of primary porcine neutrophils.

## Results

### GM1 mediates the binding of LT to neutrophils, whereas neither LT nor pSta affects neutrophil viability

**Figure 1.**
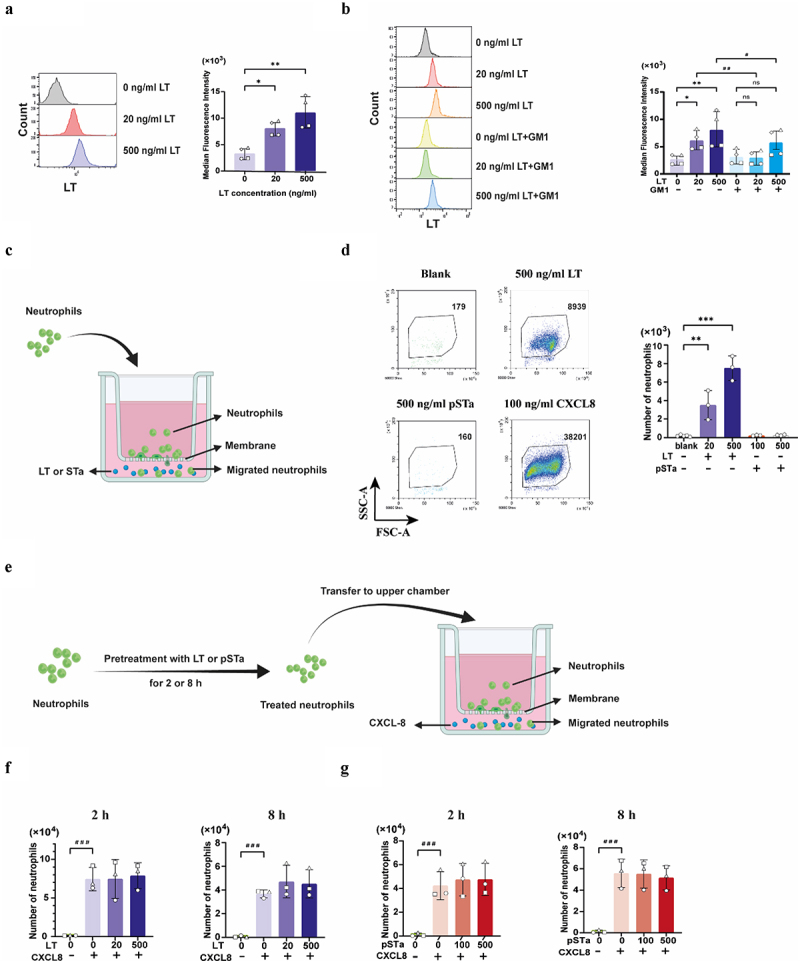
LT binds to neutrophils and triggers their migration.

To understand whether LT and pSTa can affect the effector functions of neutrophils, LT was purified from the supernatant of a porcine wild type ETEC strain^23^ (Fig. S1), while pSTa was synthesized. The bioactivity of both LT and pSTa was evidenced by their ability to increase cAMP and cGMP levels in gut epithelial cells, respectively (Fig, S2), and by their capacity to induce swelling in gut organoids (Fig. S3, movie S1–3). A key step in the enterotoxicity of LT is its binding to GM1^4,24^ however, whether LT binds to the membrane of neutrophils in a GM1-dependent manner is currently unknown. Thus, we set out to test this by incubating neutrophils with LT and evaluating its binding to the neutrophil membrane. As shown in Figure 1a, a dose-dependent increase in membrane bound LT was observed upon incubation of neutrophils with LT, consistent with confocal microscopy images (Fig. S4). Pre-treating LT with GM1 inhibited the binding of LT to the neutrophils, confirming that the binding of LT to neutrophils was indeed mediated by GM1 (Figure 1b).

(a) Neutrophils (2×10^5^) were incubated with 0, 20 or 500 ng/mL LT for 1 h at 4°C. The binding of LT to the neutrophil membrane was analyzed by immunostaining and flow cytometry. (b) Pre-incubation of 0, 25 or 500 ng/mL LT with 1 μg/mL GM1 for 2 h inhibited the binding of LT to neutrophils. (c) Scheme of the transwell migration assay. Neutrophils (3×10^5^) were added to the upper chamber of the transwells (pore size 3 μm) and 0, 20 and 500 ng/mL LT or 0, 100 and 500 ng/mL pSTa was added to the lower chamber. Upon incubation for 3 h at 37°C, the number of neutrophils in the lower chamber was assessed by flow cytometry. (d) Representative dot plots of neutrophil migration toward 500 ng/mL LT, 500 ng/mL pSTa and 100 ng/mL CXCL-8 for 3 h. Quantification analysis of migrated neutrophils toward 0, 20 and 500 ng/mL LT or 0, 100 and 500 ng/mL pSTa, added to the lower chamber. (e) Scheme of the transwell migration assay. Neutrophils (3×10^5^) were pretreated with 0, 20 and 500 ng/mL LT or 0, 100 and 500 ng/mL pSTa for 2 or 8 h and then were added to the upper chamber, while 100 ng/mL CXCL-8 was added to the lower chamber. Upon incubation for 3 h at 37°C, the number of neutrophils in the lower chamber was assessed by flow cytometry. (f) Quantification analysis of migrated neutrophils toward 100 ng/mL CXCL-8 after treatment with 0, 20 and 500 ng/mL LT for 2 or 8 h or (g) 0, 100 and 500 ng/mL pSTa for 2 or 8 h. *n* = 4 individual blood donors. The error bars represent the mean ± SD. The data of the binding assay were analyzed with a non-parametric Friedman test. The migration assay data were analyzed with One-way ANOVA with a posthoc Tukey test. A paired Student's T test was used to compare two groups with or without GM1 or CXCL8. *, ^#^ *p* < 0.05; **, ^##^ *p* < 0.01, ns: not significant.

To eliminate pathogens, neutrophils must however first migrate to the site of infection. Thus, we investigated whether LT and pSTa might affect the migration of neutrophils. To exclude a potential effect of the enterotoxins LT and pSTa on the viability of neutrophils, a cell viability assay was performed. Both LT and pSTa up to 500 ng/mL did not decrease the viability of neutrophils upon incubation for 2 or 8 h (Fig. S5). We then examined whether LT and pSTa could induce neutrophil migration in a transwell migration assay. To this end, neutrophils were added to the apical compartment of the transwells, while LT or pSTa were added to the basolateral compartment (Figure 1c). Upon incubation, neutrophils that migrated to the basolateral compartment were counted by flow cytometry. While pSTa did not influence neutrophil migration, LT clearly induced migration of neutrophils in a dose-dependent manner (Figure 1d). We also assessed whether LT and pSTa might influence the migration induced by CXCL-8, a potent chemotactic cue for neutrophils.^25^ Neutrophils were pretreated with LT or pSTa for 2 and 8 h and then added to the apical compartment of the transwells, while CXCL-8 was added to the basolateral compartment (Figure 1e). After incubation, neutrophils that migrated to the basolateral compartment were counted by flow cytometry. The results indicated that LT and pSTa did not affect the CXCL-8-dependent migration of neutrophils (Figure 1f,g).

### LT affects neutrophil phagocytosis without influencing ROS production, while pSta has no impact on neutrophil function

**Figure 2.**
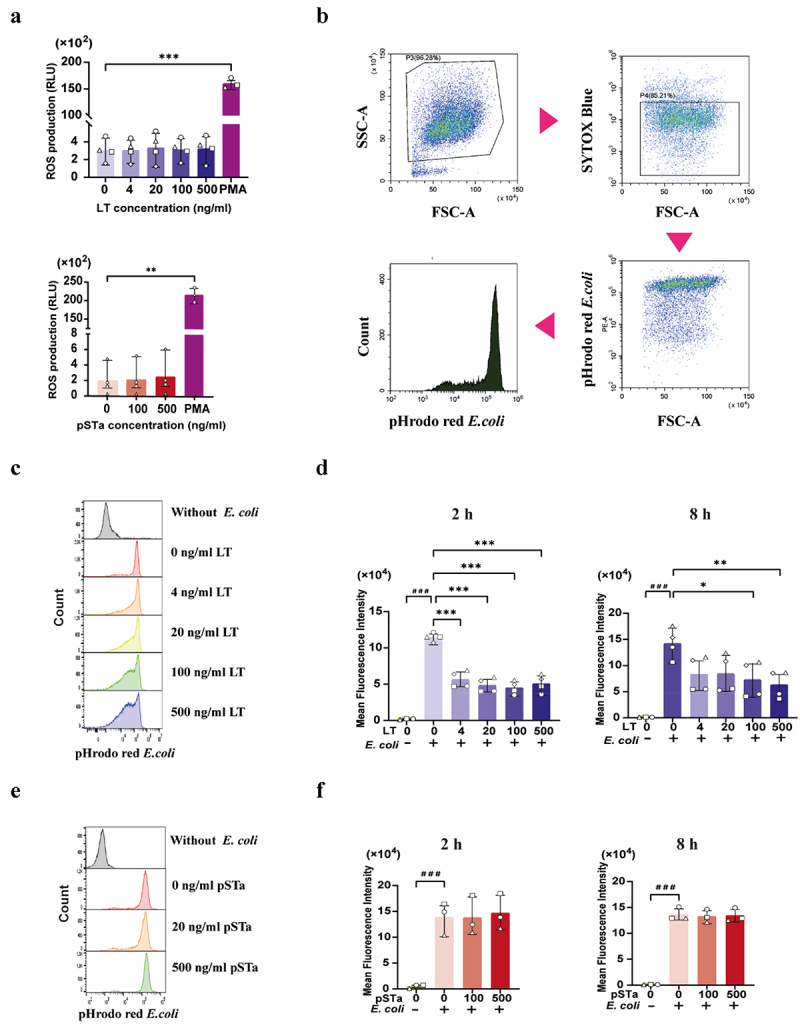
LT, but not pSta, decreases the phagocytosis ability of neutrophils.

Neutrophils have potent antimicrobial effector functions, such as the production of ROS and phagocytosis. We next wondered whether LT and pSTa might also affect neutrophil effector functions. Neutrophils can generate reactive oxygen species (ROS) to destroy pathogens.^16,26^ However, to date it is still unclear whether LT and pSTa have an impact on neutrophil ROS production. Figure 2a clearly show that neither LT nor pSTa affected ROS production by neutrophils. Furthermore, treating neutrophils with LT or pSTa for 2 and 8 h did not influence ROS production by neutrophils induced by phorbol myristate acetate (PMA), a potent activator of neutrophils (Fig. S6).

(a) Neutrophils (2×10^5^) were incubated with 0–500 ng/mL LT or 0–500 ng/mL pSTa for 2 h at 37°C and their ROS production was measured in a chemiluminescence assay. RLU: relative light units. (b) The gating strategy to assess uptake of pHrodo^TM^ red *E. coli* by flow cytometry. (c) Representative histograms showing the phagocytosis of pHrodo^TM^ red *E. coli* by neutrophils upon incubation with LT or (e) pSTa for 8 h at the indicated concentrations. (d) Phagocytosis of pHrodo^TM^ red *E. coli* by neutrophils (2×10^5^) after treatment with LT or (f) pSTa for 2 or 8 h at the indicated concentrations. *n* = 3 to 4 individual blood donors. The bars represent the mean ± SD. Data were analyzed with One-way ANOVA with a post hoc Tukey test to compare LT or pSTa treatment groups to the control group. **p < 0.01, ***p < 0.001. A paired Student's T test was used to compare two groups with or without *E. coli*. ^###^*p* < 0.001.

In addition to ROS production, phagocytosis by neutrophils is a crucial step to clear microbes, dead cells and damaged tissues.^16,27^ Here, pHrodo-labeled *E. coli* was used to evaluate the potential effects of LT and pSTa on the phagocytosis of bacteria by neutrophils. As shown in Figure 2b-d, LT significantly reduced the phagocytosis of *E. coli* by neutrophils, even at a concentration as low as 4 ng/mL. In contrast, pSTa did not change the ability of neutrophils to phagocytose *E. coli* (Figure 2e,f).

### LT downregulates the cell surface expression of CD11b by neutrophils

**Figure 3.**
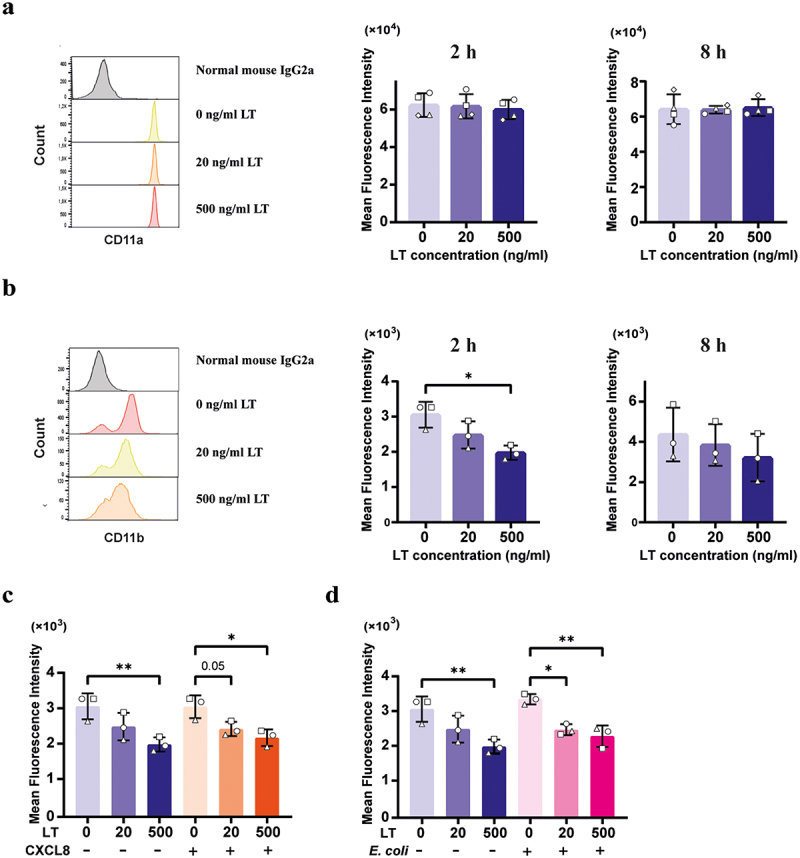
LT downregulated CD11b cell surface expression of neutrophils.

In neutrophils, β2 integrins, composed of CD18 and CD11a or CD11b, regulate different cellular processes, such as cell migration, ROS production and phagocytosis.^28,29^ Given that LT induced neutrophil migration and decreased phagocytosis of bacteria by neutrophils, we investigated whether LT might induce changes in the cell surface expression levels of CD11b or CD11a of neutrophils. While CD11a expression levels were not altered in LT-stimulated neutrophils (Figure 3a), a 2 h stimulation of neutrophils with 500 ng/mL LT downregulated the expression of CD11b (Figure 3b). Furthermore, LT also downregulated CD11b surface expression in the presence of CXCL-8 (Figure 3c) and *E. coli* (Figure 3d).

Neutrophils (2×10^5^) were first pretreated with 0, 20 and 500 ng/mL LT for 2 or 8 h at 37°C. The expression of CD11a (a) and CD11b (b) was analyzed by flow cytometry. Neutrophils (2×10^5^) were first pretreated with 0, 20 and 500 ng/mL LT for 2 h at 37°C and then incubated with 100 ng/mL CXCL-8 (c) or 6 × 10^6^ pHrodo^TM^ red *E. coli* (d) for another 2 h. The expression of CD11a was analyzed by flow cytometry. *n* = 3 to 4 individual blood donors. The bars represent the mean ± SD. Data were analyzed with One-way ANOVA with a posthoc Tukey test to compare LT treatment groups to the control group. *p < 0.05, **p < 0.01.

### LT induces secretion of cytokines and chemokines by neutrophils

**Figure 4.**
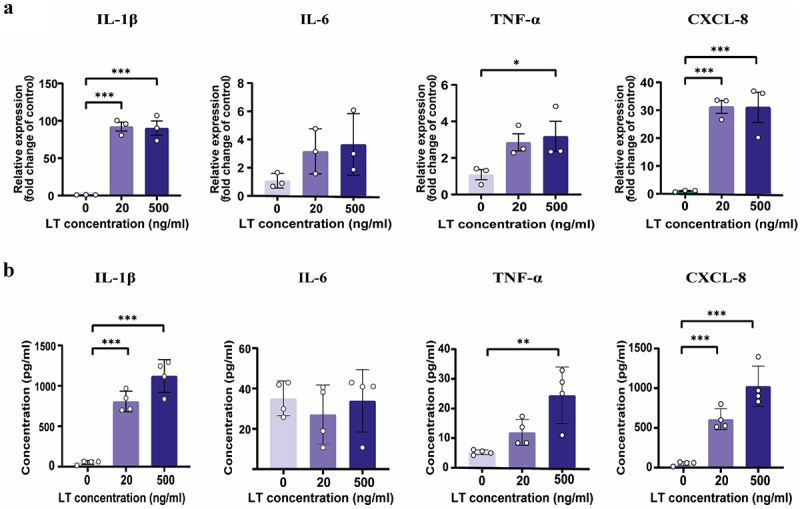
Neutrophils respond to LT by increased production of pro-inflammatory cytokines and chemokines.

Upon recognition of pathogens, neutrophils secrete pro-inflammatory mediators to inform neighboring cells and attract other immune cells.^18^ To address whether LT affects the ability of neutrophils to produce these mediators, the mRNA expression of the pro-inflammatory cytokines IL-1β, IL-6 and TNF-α as well as the chemokine CXCL-8 by porcine neutrophils was assessed upon stimulation with LT. Upon a 2-hour exposure to LT, neutrophils upregulated the mRNA expression of IL-1β, TNF-α and CXCL-8 as compared to control cells (Figure 4a). As shown in Figure S7, LT also increased the mRNA expression of the chemokines CCL3 and CCL5 but decreased CCL2 transcript levels. Similar to the transcript levels, neutrophils secreted more IL-1β, TNF-α and CXCL-8 in response to LT, while IL-6 secretion levels were not influenced by LT (Figure 4b).

(a) Neutrophils (2×10^6^) were treated with LT at the indicated concentrations for 2 h. IL-1β, IL-6, TNF-α and CXCL-8 transcript levels were evaluated by qPCR. (b) IL-1β, IL-6, CXCL-8 and TNF-α secretion levels were measured by ELISA in the culture supernatant of neutrophils (2×10^6^) after treatment with LT at the indicated concentrations for 8 h. *n* = 3 to 4 individual blood donors. The bars represent the mean ± SD. Data were analyzed with One-way ANOVA with a posthoc Tukey test to compare LT treatment groups to the control group. *p < 0.05; **p < 0.01, ***p < 0.001.

### LT promoted neutrophils to generate NETs in vitro.

**Figure 5.**
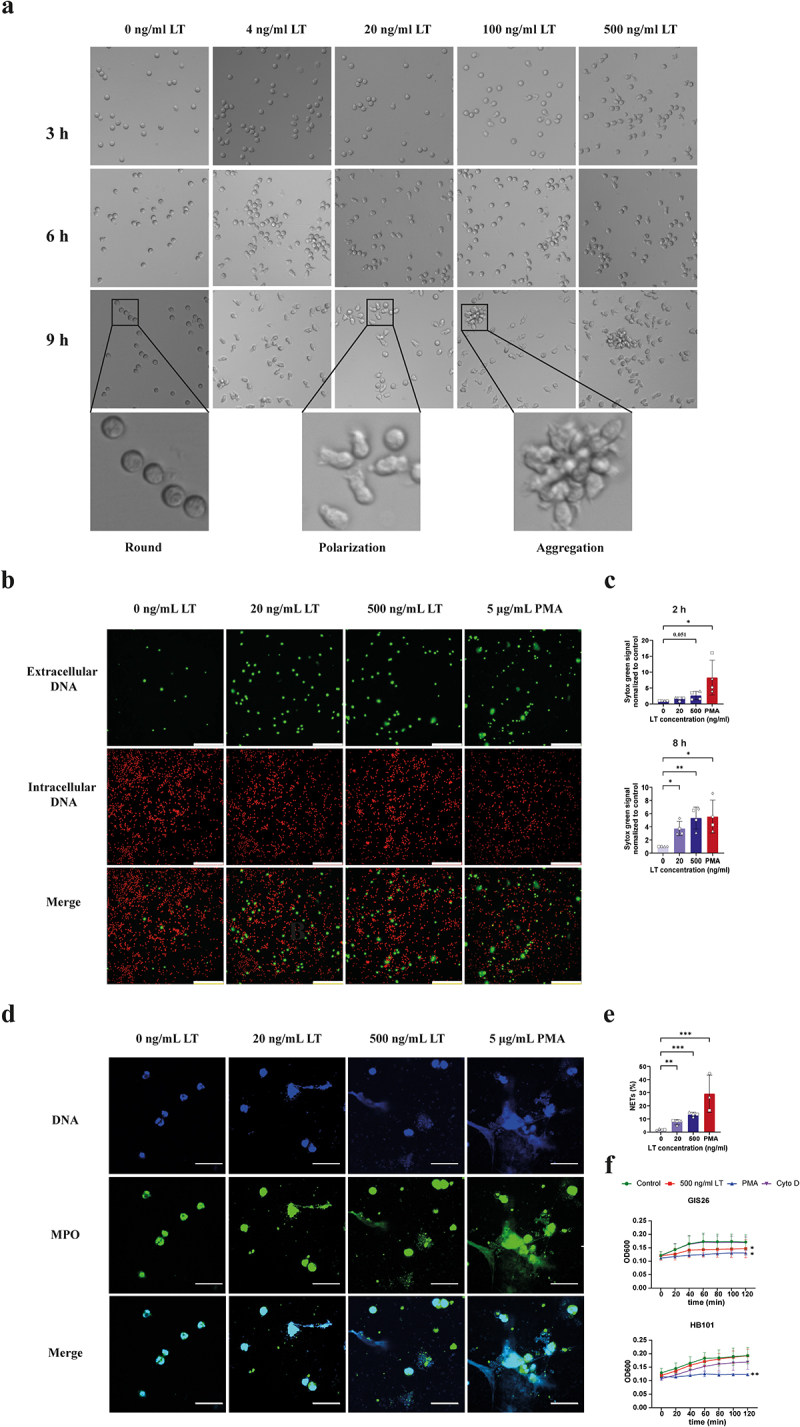
LT promoted neutrophils to generate NETs *in vitro*.

A crucial effector function of neutrophils is the formation of neutrophil extracellular traps (NETs). These NETs are formed by expulsion of DNA and enzymes from the neutrophil.^16^ We thus examined NETs formation after LT treatment. The latter changed the morphology of the neutrophils from a round to an elongated shape in a time- and dose-dependent manner (Figure 5a). This change in morphology resembles NETs formation. To confirm this, we quantified the presence of extracellular DNA in LT-treated neutrophils. As shown in Figure 5b, LT treatment increased the presence of extracellular DNA on neutrophils in a dose-dependent manner (Figure 5b,c). Furthermore, staining for the NET marker myeloperoxidase (MPO) further confirmed the generation of NETs by LT treated neutrophils (Figure 5d,e). While NETs are known to kill extracellular bacteria, previous studies have demonstrated that pathogenic bacteria can evade entrapment and killing by NETs.^30^ To understand how LT-induced NETs affect the growth of bacteria, we cultured an *E. coli* lab strain (HB101) and an ETEC strain (GIS26) in the presence of neutrophils after NETosis induction. As shown in Figure 5f, both PMA- and LT-induced NETs inhibited the growth of the ETEC strain GIS26, while only PMA-induced NETs inhibited the growth of the *E. coli* strain HB101.

(a) Neutrophils (2×10^5^) were incubated with varying concentrations of LT (0, 4, 20, 100 and 500 ng/mL) at 37°C. The morphology of the neutrophils was observed under 200× magnification at 3, 6 and 9 h. Representative images of neutrophil morphology (*n* = 4). (b) Neutrophils (2×10^5^) were incubated with 0, 20 and 500 ng/mL LT for 8 h. Extracellular DNA was stained with Sytox Green and intracellular DNA with Syto Red. Representative images are shown (*n* = 4). Scale bars, 100 μm. (c) Quantification of fluorescence intensity of Sytox green signal. (d) Neutrophils (2×10^6^) were incubated with 0, 20 and 500 ng/mL LT for 8 h and stained for MPO. Representative confocal microscopy images of NETs (DNA: blue; MPO, green). Scale bars, 10 μm. (e) Quantification of the percentage of NETs in all observed neutrophils. (f) Neutrophils were pretreated with 500 ng/mL LT or 5 μg/mL PMA for 8 h, or pretreated with 10 µg/mL cytochalasin D (Cyto D) for 20 min. The *E. coli* strain HB101 and the ETEC strain GIS26 (2×10^6^ CFU/ml) were grown for 2 h at 37°C in the presence of neutrophils. The OD_600_ was measured every 20 min. *n* = 3 to 6 individual blood donors. The bars represent the mean ± SD. Data of NETs formation were analyzed with one-way ANOVA with a posthoc Tukey test to compare LT treatment groups to the control group. *p < 0.05, **p < 0.01, ***p < 0.01. A paired Student's T test was used to compare two groups with or without PMA. ###, *p* < 0.001. Data of antimicrobial activity of NETs were analyzed with a paired Student’s T test to compare treatment groups to the control group at the 120 min timepoint. *p < 0.05; **p < 0.01.

### LT augments intracellular cAMP levels of neutrophils and activates PKA signaling.

**Figure 6.**
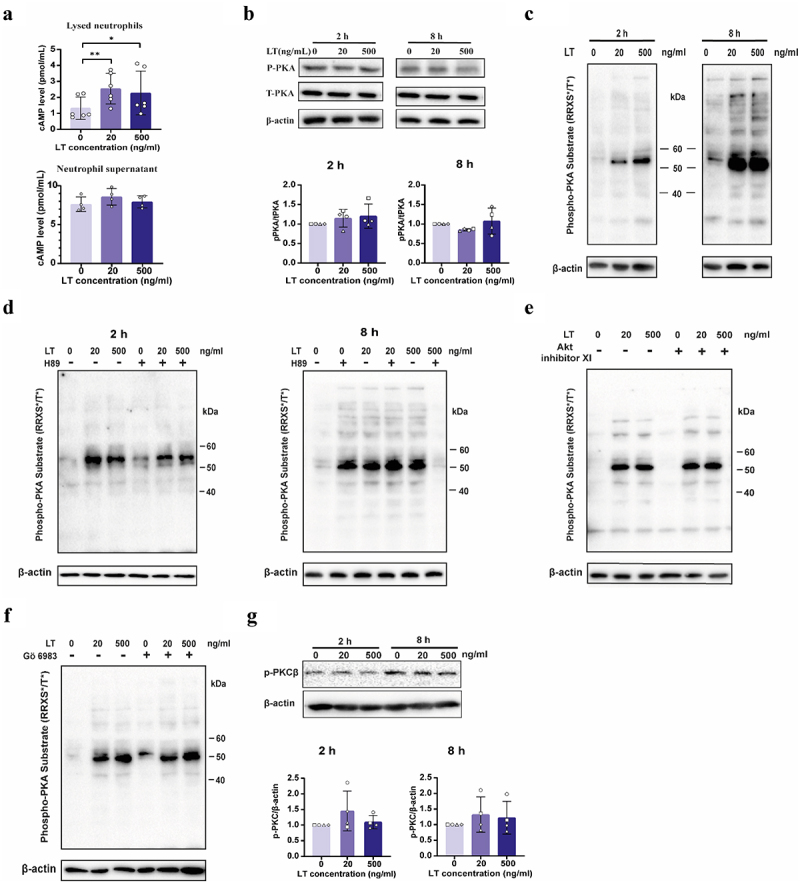
LT induced activation of the cAMP/PKA signaling pathway in porcine neutrophils.

In intestinal epithelial cells, binding of LT to GM1 triggers uptake of LT, which in turn elicits increased cAMP levels and activation of PKA.^4^ Whether LT also activates the cAMP/PKA pathway in neutrophils was still unknown. Interestingly, we found that intracellular cAMP levels in neutrophils were elevated upon stimulation with LT for 90 min, while extracellular cAMP levels were not affected (Figure 6a). Subsequently, we examined additional PKA activation by quantifying the levels of both PKA and phosphorylated PKA. The results showed that LT did not change total PKA levels, nor increased the phosphorylation of PKA in porcine neutrophils (Figure 6b). This seems to indicate that the LT-induced increase in cAMP levels suffices to activate PKA in neutrophils. cAMP-dependent PKA belongs to the Arg-directed kinases or AGC kinase family.^31^ These serine/threonine protein kinases share a common recognition site characterized by the presence of an Arg at position −3 relative to the Ser/Thr. To investigate the potential activation of PKA upon LT stimulation of neutrophils, a phospho-PKA substrate antibody was used to evaluate the presence of phosphorylated PKA substrates. Upon 2 and 8 h stimulation of neutrophils with LT the levels of proteins phosphorylated at the AGC kinase recognition site were increased (Figure 6c). In an effort to confirm a role for PKA, neutrophils were pre-treated with the PKA inhibitor H89. The results indicated that PKA inhibition reduced the levels of phosphorylated proteins induced by stimulation of neutrophils with LT for 2 h. This reduction was not observed when neutrophils were stimulated with LT for 8 h (Figure 6d, S8). The AGC kinase family contains many members. Since inhibiting PKA did not completely prevent phosphorylation of target proteins, other kinases such as Akt or PKC might also be involved. To address this possibility, we pre-treated the neutrophils with the Akt inhibitor XI or the PKC inhibitor Go6983 and then evaluated the levels of phosphorylated substrates in the neutrophils upon LT stimulation. Inhibition of Akt and PKC did not prevent the phosphorylation of substrates triggered by LT (Figure 6e-f, S8). Notably, as PKC regulates migration and phagocytosis of neutrophils, we further assessed PKC activation. Western blot analysis showed that stimulation with LT did not affect the levels of phosphorylated PKC (Figure 6g).

(a) The cAMP level in lysed neutrophils and neutrophil supernatant after treatment with 0, 20 and 500 ng/mL LT for 90 min. (b) The total PKA and phosphorylated PKA levels in neutrophils after treatment with LT for 2 or 8 h. (c) Representative immunoblots of substrates phosphorylated by PKA (phospho-PKA) in neutrophils after treatment with LT for 2 or 8 h. (d) Representative immunoblots of phospho-PKA substrates in neutrophils pre-treated with 10 μM H89 for 2 h, and then treated with LT for 2 or 8 h. (e) Representative immunoblot of phospho-PKA substrates in neutrophils pre-treated with the Akt inhibitor XI (10 μM) or (f) the PKC inhibitor Go6983 (10 μM) for 2 h, and then treated with LT for another 2 h. (g) The expression of β-actin and phosphorylated PKC in neutrophils after treatment with LT for 2 h or 8 h. *n* = 3 to 4 individual blood donors. The bars represent the mean ± SD. Data were analyzed with One-way ANOVA with a posthoc Tukey test to compare LT treatment groups to the control group. *p < 0.05, ***p < 0.01.

### ERK1/2 is activated upon LT induced activation of PKA signaling.

**Figure 7.**
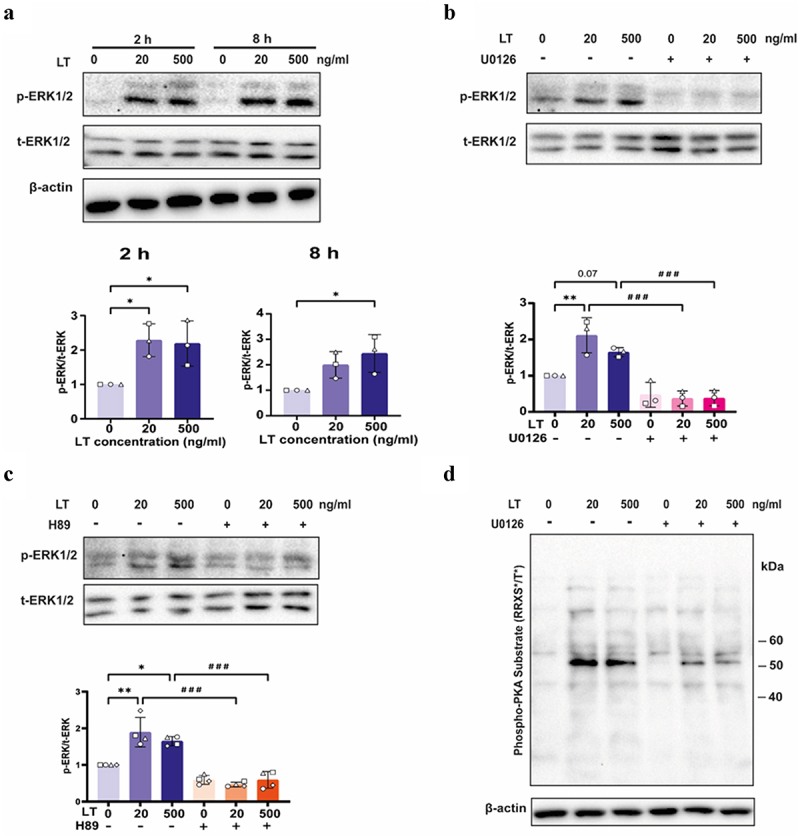
LT induced ERK1/2 phosphorylation in neutrophils.

In addition to the cAMP/PKA pathway, the activation of the ERK1/2 pathway in intestinal epithelial cells and dendritic cells has also been reported in studies investigating LT.^32,33^ Guided by these previous studies, we examined the activation status of ERK1/2 in neutrophils. Interestingly, western blot analysis indicated that LT induced ERK1/2 phosphorylation (Figure 7a), which was inhibited upon pretreatment of the neutrophils with the ERK1/2 inhibitor U0126 (Figure 7b). Previous research showed a crosstalk between the cAMP/PKA and ERK1/2 signaling pathways.^34,35^ Our results showed that the PKA inhibitor H89 inhibited the phosphorylation of ERK1/2 (Figure 7c). In addition, inhibition of ERK1/2 activity resulted in decreased levels of phosphorylated PKA substrates in neutrophils induced by LT (Figure 7d).

(a) Total ERK1/2 and phosphorylated ERK1/2 levels in neutrophils after treatment with LT for 2 or 8 h. (b) Total ERK1/2 and phosphorylated ERK1/2 levels in neutrophils pre-treated with 10 μM U0126 for 2 h, and then treated with LT for 2 h. (c) Protein levels of total ERK1/2 and phosphorylated ERK1/2 in neutrophils pre-treated with 10 μM H89 for 2 h, and then treated with LT for 2 h. (d) Representative immunoblot of phospho-PKA substrates in neutrophils pre-treated with 10 μM U0126 for 2 h, and then treated with LT for another 2 h. *n* = 3 to 4 individual blood donors. The bars represent the mean ± SD. Data were analyzed with One-way ANOVA with a posthoc Tukey test to compare LT treatment groups to the control group. *p < 0.05, **p < 0.01. A paired Student's T test was used to compare two groups with or without inhibitors. ###, < 0.01.

### PKA and ERK1/2 signaling regulate neutrophil effector functions triggered by LT

**Figure 8.**
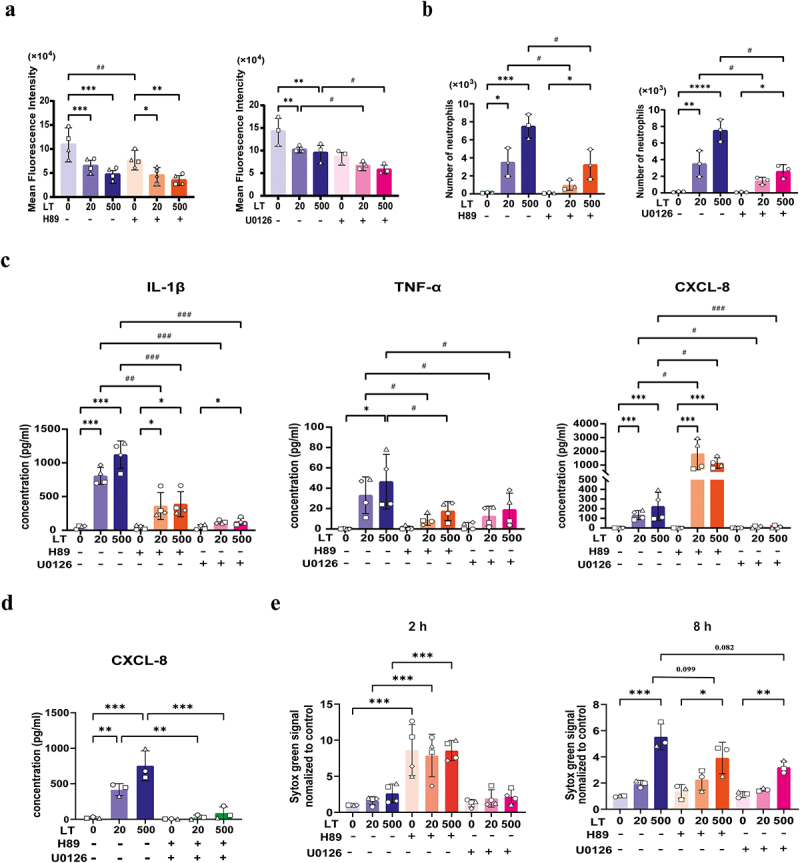
Inhibition of PKA and ERK1/2 affected migration and inflammatory factor production induced by LT.

Our results demonstrated that LT reduced phagocytosis of *E. coli* by neutrophils, triggered neutrophil migration and induced the production of inflammatory mediators by neutrophils. Concurrently, we showed that LT activated the cAMP/PKA pathway and ERK1/2 pathway in neutrophils. To determine whether the observed impact of LT on these effector functions correlates with the activation of these two pathways, neutrophils were pretreated with the PKA inhibitor H89 or the ERK1/2 inhibitor U0126. Upon LT stimulation, phagocytosis, migration, cytokine secretion and NET formation were assessed. Our findings demonstrated that H89 reduced the phagocytic activity of neutrophils in the absence of LT, while U0126 did not affect neutrophil phagocytosis (Figure 8a). Inhibiting PKA (H89) or ERK1/2 (U0126) signaling did not prevent the LT-induced reduction of bacterial phagocytosis by neutrophils. In contrast, pretreatment of neutrophils with H89 and U0126 decreased migration of neutrophils induced by LT, indicating that both PKA and ERK1/2 activation play an important role in LT-induced neutrophil migration (Figure 8b). We also investigated whether inhibiting PKA and ERK1/2 signaling might affect the LT-induced secretion of IL-1β, CXCL-8, and TNF-α. As shown in Figure 8c, pretreatment of neutrophils with H89 and U0126 significantly inhibited the LT-induced secretion of IL-1β and TNF-α by neutrophils. Of note, inhibiting PKA and ERK1/2 had opposing effects on the LT-mediated CXCL-8 secretion by neutrophils. While pretreatment with the PKA inhibitor H89 led to a significant increase in CXCL-8 secretion, pretreatment with the ERK1/2 inhibitor U0126 significantly decreased this. Furthermore, pretreatment of neutrophils with both H89 and U0126 resulted in nearly complete inhibition of the CXCL-8 secretion induced by LT (Figure 8d). Finally, we assessed whether PKA and ERK1/2 are involved in NET formation upon LT treatment of neutrophils. Our results showed that pretreatment with H89 or U0126 decreased NETs generation induced by LT (Figure 8e). Altogether, these data point to an interaction between LT-induced neutrophil effector functions and the activation of the cAMP/PKA/ERK pathway.

(a) Neutrophils (2×10^6^) were pre-incubated with DMSO, 10 μM H89 or 10 μM U0126 for 2 h, and then treated with LT for another 2 h. Phagocytosis of pHrodo^TM^ red *E. coli* by neutrophils was assessed by flow cytometry. (b) Neutrophils (2×10^6^) were incubated with DMSO, 10 μM H89 or 10 μM U0126 for 2 h and neutrophil migration induced by LT was evaluated in a transwell assay as shown in Figure 1c. (c) Neutrophils (2×10^6^) were pre-incubated with DMSO, 10 μM H89 or 10 μM U0126 for 2 h, and then treated with LT for another 8 h. IL-1β, TNF-α and CXCL-8 secretion levels were determined in neutrophil supernatant by ELISA. (d) Neutrophils (2×10^6^) were pre-incubated with DMSO or 10 μM H89 and 10 μM U0126 for 2 h, and then treated with LT for another 8 h. CXCL-8 secretion levels were determined in neutrophil supernatant by ELISA. (e) Neutrophils (2×10^5^) were pre-incubated with DMSO, 10 μM H89 or 10 μM U0126 for 2 h, and then treated with LT for another 2 or 8 h. NETs formation was determined by measuring the release of extracellular DNA. *n* = 3 to 4 individual blood donors. The bars represent the mean ± SD. Data were analyzed with One-way ANOVA with a posthoc Tukey test to compare LT treatment groups to the control group. *p < 0.05, **p < 0.01, ***p < 0.001. A paired Student's T test was used to compare two groups with or without inhibitors. ^#^*p* < 0.05, ^##^*p* < 0.01, ^###^*p* < 0.001.

## Discussion

**Figure 9.**
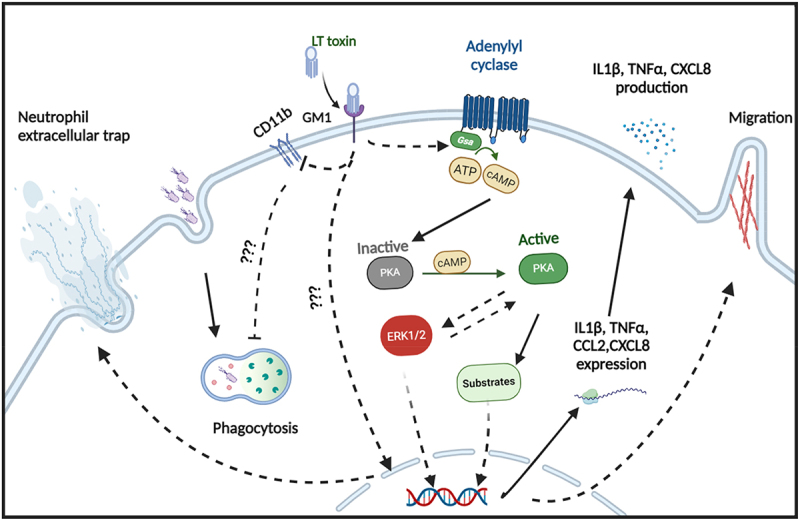
Graphical summary of the results. The full lines indicate direct effects, while the dashed lines indicate indirect effects of LT on neutrophils.

ETEC causes diarrhea in humans and livestock species.^4^ While the pathways that finally result in diarrhea, triggered by the ETEC enterotoxins LT and ST in enterocytes, are well known, knowledge on the impact of these enterotoxins on neutrophils was completely lacking.^6^ As innate immune cells, neutrophils play a critical role in clearing bacterial infections and contribute to the initiation and regulation of adaptive immunity.^16^ Here, we addressed this knowledge gap and investigated whether LT and pSTa influence the effector functions of porcine neutrophils. Our findings revealed that pSTa did not affect neutrophil effector functions. In contrast, we showed that LT reduced the phagocytic capacity of neutrophils, increased neutrophil migration and NET formation and augmented the secretion of IL-1β, CXCL-8, and TNF-α, while ROS production was unaffected by this enterotoxin. Moreover, we demonstrate that the LT-induced migration and cytokine secretion by neutrophils can be attributed to the activation of a cAMP/PKA/ERK signaling pathway in these cells (Figure 9).

In intestinal epithelial cells, LT binds to GM-1, resulting in its internalization. This uptake leads to a proteolytic cleavage of the A subunit into the A1 and A2 domains. The A1 domain contains the catalytic activity and irreversibly activates adenylate cyclase, which converts ATP in cAMP. When cAMP binds to the regulatory subunit of PKA, it triggers the dissociation of the catalytic subunits, leading to the phosphorylation of downstream target proteins and transcription factors.^31,36^ Our results indicate that a similar mechanism occurs in neutrophils. GM1 is present in the membrane of human neutrophils^37^ and adding GM-1 to LT blocked its binding to porcine neutrophils, suggesting that GM1 may serve as a receptor for LT on neutrophils. Notably, we observed an increase in intracellular cAMP levels in neutrophils after LT treatment, leading to PKA activation and subsequent phosphorylation of PKA substrates. In addition to the cAMP/PKA pathway, activation of ERK1/2 by LT has also been reported in epithelial cells as well as in dendritic cells.^32,33,38,39^ Similar to these studies, our results also show that LT activates the ERK1/2 signaling pathway in neutrophils. Like other studies showing a multifaceted interplay between these two signaling pathways,^34,35,40^ our data also point toward a crosstalk between the PKA and ERK1/2 signaling pathways, since the ERK1/2 inhibitor attenuated LT-induced activation of the cAMP/PKA pathway, and the PKA inhibitor reciprocally curtailed LT-induced activation of ERK1/2. Further research is needed to comprehensively elucidate the role of this interplay in the impact of LT on neutrophil effector functions.

Migration of neutrophils to inflamed or infected sites is regulated by chemokine gradients secreted by cells within or surrounding the infection site.^25^ Interestingly, LT can induce the secretion of CXCL-8 and IL33 by epithelial cells,^12,33,41,42^ suggesting that LT might affect the migration of neutrophils. For instance, intradermal administration of LT to mice recruits neutrophils to the skin.^43^ In addition to responding to chemokine gradients, a recent study showed that neutrophils can directly recognize and respond to bacterial toxins from *S. aureus* to migrate to the infection site.^44^ In our study, LT directly induced neutrophil migration, suggesting that neutrophil migration upon recognition of secreted bacterial products might be conserved across tissues and species. This migration of neutrophils toward LT involved PKA and ERK signaling. While ERK1/2 signaling has a known role in neutrophil migration,^45,46^ the impact of intracellular cAMP levels and PKA activation on neutrophil chemotaxis remains a topic of debate. Some studies suggested that the cAMP/PKA pathway plays a crucial role in neutrophil migration,^47,48^ while others proposed that its activation may hinder chemotaxis.^45,49^ The reason for this discrepancy remains unknown and further work will be necessary to fully define the role of these signaling pathways in neutrophil migration.

At the site of infection, neutrophils execute several antimicrobial activities to clear the threat, including phagocytosis and the production of superoxides.^50^ Here, we showed that neither LT nor pSTa induced ROS production by neutrophils, nor did they inhibit ROS production induced by a potent neutrophil activator (PMA). In contrast, LT diminished the ability of neutrophils to phagocytose *E. coli*, independent from PKA and ERK1/2 signaling. The reduced phagocytosis triggered by LT might be due to the reduced CD11b expression levels in LT treated neutrophils, as CD11b/CD18 plays an important role in phagocytosis by neutrophils.^51,52^ The LT-induced reduction of neutrophil phagocytosis is in line with a recent report showing that LT reduced the ability of macrophages to phagocytose ETEC, resulting in a heightened bacterial burden.^15^ These findings collectively suggest that ETEC uses LT to subvert phagocytosis by innate immune cells (neutrophils and macrophages) and as such may enhance the persistence of ETEC within the host. Notably, our findings indicated that the Akt inhibitor XI almost completely blocked neutrophil phagocytosis (Figure S9), indicating that Akt signaling plays a crucial role in regulating phagocytosis by neutrophils. Further research is needed to understand whether LT can inhibit Akt signaling to control neutrophil phagocytosis.

Neutrophils also combat pathogens by releasing NETs, a process called NETosis.^16^ NETs are web-like chromatin structures contain DNA, histones and various antimicrobial components such as antimicrobial peptides, myeloperoxidase, and cathepsin G^17^. While NETosis often leads to the death of neutrophils, sometimes neutrophils respond to pathogens with vital NETosis, in which the neutrophils stay alive, allowing them to continue performing their functions after releasing NETs.^53^ Here, we showed that LT the triggers the formation of NETs. Since neutrophil viability remained unaffected by LT treatment, this seems to indicate that LT induces NET formation via the vital NETosis pathway.^54^ Further experiments are warranted to confirm this. The mechanisms of NETs formation are incompletely understood. While some research indicates that the ROS pathway plays a critical role in NETosis, other studies have shown that vital NETosis can occur independently of ROS.^17^ In this study, LT treatment did not induce ROS production, suggesting that ROS might not be involved in the LT-induced NETs formation. While some studies pointed to a role for ERK1/2 in NETs formation,^55,56^ our results indicated that inhibition of ERK signaling did not prevent NETs formation induced by LT. This implies a role for alternate pathways in LT-induced NETs generation. Interestingly, activation of PKA can inhibit NETs formation.^57,58^ Here, we found that inhibiting PKA enhanced NETs formation in the absence of LT. This seems to indicate that PKA prevents NETosis in steady conditions and that LT activates PKA to dampen NET formation, at least temporarily. Although NETs are a defense mechanism against pathogens, some pathogenic bacteria can escape entrapment and killing by NETs and even use these NETs as a nutrient source.^59,60^ Our results showed that LT-induced NETs inhibited the growth of ETEC similarly to PMA, indicating that ETEC did not develop strategies to counteract the antimicrobial activity of NETs.

In addition to these antimicrobial activities, neutrophils can also release a spectrum of inflammatory mediators, including cytokines, chemokines, leukotrienes, and prostaglandins, which can subsequently regulate the activity of other immune cells and stromal cells.^61^ In this study, LT triggered an increased secretion of IL-1β, TNF-α and CXCL8 in neutrophils. Other innate immune cells like dendritic cells and monocytes also secrete pro-inflammatory cytokines upon contact with LT.^32,62^ When investigating the involved signaling pathways, our results illustrated that PKA and ERK1/2 signaling are involved in the LT-induced upregulation of IL-1β and TNF-α secretion by neutrophils. Although it is known that ERK activation can induce production of IL-1β and TNF-α,^63^ the influence of PKA activation on their production by neutrophils remains incompletely understood. While some studies showed that PKA activation induces production of IL-1β and TNF-α,^64,65^ others demonstrated that PKA activation inhibits IL-1β and TNF-α production.^66,67^ A possible explanation for the conflicting results may result from the different stimulus and treatment time used in the different cellular contexts. In the context of CXCL-8 secretion, our findings suggest that PKA and ERK1/2 signaling have opposing functions in the response of neutrophils to LT. More specifically, the PKA signaling cascade activated by LT controls CXCL-8 secretion by neutrophils and also activates ERK1/2, which then attenuates the magnitude of the CXCL-8 response.

Our experiments show that the enterotoxin LT affects neutrophil effector functions in part through the cAMP/PKA/ERK signaling pathway using validated inhibitors. Nevertheless, a main limitation to this study is the lack of knockout and overexpression data to elucidate the role of key proteins in these LT-induced changes in neutrophil effector functions. Neutrophils are terminally differentiated cells and do not survive long both in circulation and in culture. This short lifespan of primary neutrophils limits the feasibility of generating gene deletion and overexpression mutants in these cells with current technologies. Future research should focus on developing innovative methods and tools, like conditional neutrophil-specific transgenic pigs, to enable this research in large animal models.

In conclusion, while the heat stable enterotoxin pSTa did not affect neutrophil effector functions, ETEC-derived LT induced neutrophil migration and production of inflammatory mediators by activating the cAMP/PKA and ERK1/2 signaling pathways. This might aid in clearance of ETEC infections by neutrophils and other immune cells. However, LT shuts down neutrophil phagocytosis and dampens NET formation which may be advantageous for ETEC to establish and maintain infection in the gut.

## Materials and methods

### Enterotoxins

pSTa was synthesized by Bachem company (Bachem Holding). LT was purified from the supernatant of ETEC strain IMM07 (O147:K88, F4ac, LT^+^STa^−^STb^+^) as previously described^68^ with some modifications. The bacterial strain was grown in CAYE medium for 24 h at 37°C with vigorous stirring. Bacteria were pelleted by centrifuging the culture medium at 5000 g for 20 min at 4°C. Upon collection of the supernatant, ammonium sulfate (Sigma) was added to the supernatant until a 55% saturation level. Upon stirring overnight incubation at 4°C, the precipitated proteins were harvested by two rounds of centrifugation at 5000 g for 30 min at 4°C. The pellet was dissolved in 200 mL TEAN buffer (50 mM Tris-HCl (pH 7.5), l mM EDTA and 200 mM NaCl) and dialyzed against TEAN buffer overnight at 4°C. The resulting solution was loaded on a galactose agarose (Thermo Fisher Scientific) column at a flow rate of 0.5 mL/min. Unbound proteins were removed with 10 column volumes of TEAN buffer. The bound proteins were eluted with TEAN buffer containing 0.12 M galactose (Sigma) and then dialyzed against distilled water overnight at 4°C. Protein precipitates were removed by centrifugation at 12,000 g for 10 min, 4°C, and then pooled using a centrifugal filter (Millipore). The presence of LT in the elution fractions was determined with western blotting, while the purity of LT was determined by SDS-PAGE and a silver staining kit (Thermo Fisher Scientific). The concentration of LT was determined by GM1-ELISA using rabbit anti-heat labile toxin antibody (Abcam) as described (Wang et al., 2020). The purified LT was stored at 4°C until use.

### Isolation of neutrophils

The neutrophils were isolated from blood as described previously.^69^ In brief, peripheral blood was collected from 10 to 24-week-old pigs via the jugular vein on heparin. After mixing with the same volume of RPMI 1640 medium (Gibco), neutrophils were isolated by density gradient centrifugation on a discontinuous Percoll gradient (68% and 75%, GE Healthcare). Neutrophils were resuspended at a density of 2 × 10^6^ cells/mL in phenol red-free RPMI 1640 medium (Gibco) containing 10% fetal calf serum (FCS, Greiner) and 1% penicillin/streptomycin (Gibco). All animal experiments were approved by the animal care and ethics committee of the Faculty of Veterinary Medicine, Ghent University (EC2017/121 and EC2023/22).

### cAMP and cGMP ELISA

The bioactivity of purified LT was determined by measuring the cAMP production by IPEC-J2 cells, which was originally isolated from jejunal epithelium of neonatal piglet. IPEC-J2 cells were maintained in DMEM/F12 medium supplemented with 5% FCS, 1% penicillin-streptomycin, 1% insulin-transferrin-selenium (ITS, Sigma), 2% L-glutamine and 5 ng/mL epidermal growth factor (EGF, Gibco) and incubated at 37°C, 5% CO_2_ and 95% humidity. The bioactivity of the synthesized pSTa (Bachem) was determined by measuring the cGMP production by T84 cells which was derived from a colon carcinoma in a 72-year-old man. T84 cells were cultured in DMEM/F12 medium supplemented with 5% FCS and 1% P/S and incubated at 37°C, 5% CO_2_ and 95% humidity. IPEC-J2 cells (1 × 10^5^ cells/well) and T84 cells (2 × 10^5^ cells/well) were seeded into 24-well plates and cultured for two days to reach at least 90% confluence. LT (0, 20, 100, 500 ng/mL) was added to IPEC-J2 cells, while pSTa (0, 100 and 500 ng/mL) was added to the T84 cells. The cells were subsequently incubated for 1 h at 37°C, 5% CO_2_ and 95% humidity. After removal of the culture medium, the cells were lysed with 0.1 M HCl to stop endogenous phosphodiesterase activity. Upon centrifugation at 660 g for 10 min at room temperature to remove cellular debris, the IPEC-J2 lysate was assayed for cAMP using a Direct cAMP ELISA Kit (Enzo Life Sciences) and the T84 lysate was assayed for cGMP using a Direct cGMP ELISA Kit (Enzo Life Sciences), following the manufacturer’s guidelines.

Neutrophils were seeded at 2 × 10^6^ cells/well in 24-well plates. After 1 h incubation, LT (0, 100 and 500 ng/mL) was added to the culture medium and incubated at 37°C for 10 or 90 min. Next, the neutrophils were collected and centrifuged at 400 g for 5 min, 4°C. The supernatant was transferred into a new tube and the cells were lysed with 300 μL 0.1 M HCl. The cAMP and cGMP levels in the supernatant and cell lysates were then quantified using a Direct cAMP or cGMP ELISA Kit according to the manufacturer’s instructions.

### Swelling assay in gut organoids

Small intestinal crypts were isolated from 8-week-old piglets and maintained until enteroids could be passaged. These enteroids were then used to perform a swelling assay as previously described.^70^ Briefly, enteroid fragments were cultured in 6 μL Matrigel (growth factor reduced, Corning) containing 1 μL organoids growth medium (OGM, 1:1 mix of OGM human basal medium and organoid supplement, Stem Cell Technologies) and 10 μM Y-inhibitor (Sigma) as well as 50 μL OGM medium for two days until small enteroids developed. Then, LT (100 ng/mL) or pSTa (1 μg/mL) were added to the culture medium. The enteroids were monitored for three hours using a live-cell microscope (Olympus IX81) with controlled temperature (37 °C) and CO_2_ (5%). Five to ten enteroids were selected at random and every 10 min the enteroids were imaged. The resulting time-lapse was analyzed with ImageJ. At the 80 min and 180 min timepoint, the surface area of the enteroids was measured manually. The relative increase in surface area at the indicate timepoint was then calculated by dividing the area measured at that timepoint by the area of the initial state (*T* = 0).

### LT binding assay

The binding of LT to the membrane of neutrophils was measured by flow cytometry. Neutrophils were seeded at 2 × 10^5^ cells/well in 96-well plates and incubated at 37°C, 5% CO_2_ for 1 h. Then, neutrophils were incubated with 0, 20 or 500 ng/mL LT for another 30 min on ice. After incubation, neutrophils were washed 3 times with ice-cold PBS and were incubated for 3 h on ice with rabbit anti- *E. coli* LT polyclonal antibody (1:300, Abcam) in ice-cold PBS with 1% FCS. Upon three washes with ice-cold PBS, neutrophils were stained for 1 h on ice with PE-labeled goat anti-rabbit lgG polyclonal antibody (1:300, Invitrogen) in ice-cold PBS. Upon washing 3 times, neutrophils were stained with the viability dye Sytox^TM^ Blue (1 μM, Thermo Fisher Scientific) in PBS and analyzed by flow cytometry (Cytoflex, Beckman Coulter). The data were analyzed using CytExpert software (Beckman Coulter). For the GM1 inhibition assay, 0, 25 or 500 ng LT was preincubated with 1 μg GM1 (Sigma) for 2 h at 37°C before adding to the neutrophils.

### Cell viability assay

The viability of the neutrophils upon enterotoxin stimulation was measured by flow cytometry (Cytoflex, Beckman Coulter). Neutrophils were seeded at 2 × 10^5^ cells/well in 96-well plates and incubated at 37°C and 5% CO_2_ for 1 h. Then, neutrophils were treated with various concentrations of LT (0, 4, 20, 100 and 500 ng/mL) or pSTa (0, 100, 200, 300, 400 and 500 ng/mL) for another 4 or 10 h. After toxin treatment, neutrophils were harvested, transferred to 96-well V-bottom plates and stained with 1 μg/mL propidium iodide (PI, Sigma) on ice for 5 min. Upon doublet discrimination, neutrophils were selected based on their FSC-A/SSC-A properties. Live/dead discrimination was performed based on PI staining using a minimal event count of 10,000 neutrophils. The data were analyzed using CytExpert software (Beckman Coulter).

### Analysis of reactive oxygen species production

A luminol chemiluminescence assay was performed to evaluate the ROS production by enterotoxin-stimulated neutrophils as described previously.^69^ Neutrophils (2×10^5^ cells/well) were seeded in a 96-well white microplate and incubated for 1 h at 37°C, 5% CO_2_. The culture medium was then replaced by luminol buffer (100 μg/mL) and the background levels were measured. Then, LT (0, 32, 160, 800, 4000 ng/mL) or pSTa (0, 800 and 4000 ng/mL) was added and the chemiluminescence was continuously measured every 5 min for a period of 2 h at 37°C using a microplate reader (MTX lab system). Neutrophils stimulated with 50 μg/mL PMA (Sigma) were used as a positive control. To determine the effect of LT and pSTa on the ROS production induced by PMA, the neutrophils were pretreated with LT (0, 4, 20, 100, 500 ng/mL) or pSTa (0, 4, 20, 100, 500 ng/mL) for 2 or 8 h and then stimulated with PMA. Chemiluminescence was monitored as described above.

### Chemotaxis assay

Neutrophil chemotaxis was assessed using polycarbonate transwell inserts (6.5 mm diameter, 3 μm pore size, Falcon). To detect neutrophil migration induced by LT or pSTa, neutrophils (3×10^5^ cells) were added to the upper chamber and culture medium containing LT (20 and 500 ng/mL) or pSTa (100 and 500 ng/mL) was added to the bottom chamber. Culture medium without enterotoxin was used as a negative control, while recombinant porcine CXCL-8 (100 ng/mL; R&D systems) was used as a positive control. Upon 3 h, neutrophils in the lower chamber were collected and stained with propidium iodide (1 μg/mL in PBS). The number of cells was determined by flow cytometry (Beckman Coulter), and the data were analyzed using CytExpert software (Beckman Coulter). To evaluate whether LT and pSTa influence the migration of neutrophils induced by CXCL-8, neutrophils (3×10^5^) were pretreated with various concentrations of LT or pSTa at 37°C, 5% CO_2_ for 2 or 8 h. The pretreated neutrophils were then transferred to the upper chamber, and culture medium containing 100 ng/mL CXCL-8 was added to the bottom chamber. Culture medium without CXCL-8 was used as a control. After 3 h of incubation, the number of neutrophils in the lower chamber was determined as described above. To evaluate the role of PKA or ERK1/2 signaling in LT-induced neutrophil chemotaxis, neutrophils were preincubated with diluent control (DMSO), the PKA inhibitor H89 (10 μM, Sigma) or the ERK1/2 inhibitor U0126 (10 μM, Cell signaling Technology) for 2 h at 37 before conducting the chemotaxis assay. The used concentrations of H89 and U0126 did not affect neutrophil viability.

### Phagocytosis assay

Neutrophils (2 × 10^5^ cells/well) were pretreated with LT (0, 4, 20, 100, 500 ng/mL) or pSTa (0, 100 and 500 ng/mL) for 2 or 8 h. After incubation, pHrodo^TM^ red *E. coli* (6 × 10^6^ particles, Thermo Fisher Scientific) were added to the cells and incubated at 37°C for 2 h. Subsequently, neutrophils were collected and washed 3 times with cold PBS to remove unbound particles. Finally, neutrophils were stained with Sytox^TM^ Blue (1 μM, Thermo Fisher Scientific) and analyzed by flow cytometry (Cytoflex, Beckman Coulter). The data were analyzed using CytExpert software (Beckman Coulter). For the inhibition assay, the neutrophils were preincubated with 10 μM H89 or U0126 for 2 h at 37°C before LT was added to neutrophils.

### Analysis of CD11a and CD11b expression

To check the cell surface expression levels of CD11a and CD11b by neutrophils, neutrophils (2×10^5^ cells/well) were treated with LT (0, 20 and 500 ng/mL) for 2 or 8 h. After this treatment, the neutrophils were stained with FITC-conjugated anti-porcine CD11a antibody (1:10 dilution, Bio-Rad) or FITC-conjugated anti-porcine CD11b (1:10 dilution, Abcam) antibody in PBS + 1%FCS for 40 min on ice. Upon washing to remove any unbound antibodies, the neutrophils were stained with 1 μM Sytox^TM^ Blue and measured using flow cytometry (Cytoflex, Beckman Coulter). The data were analyzed using CytExpert software (Beckman Coulter).

To check the cell surface expression levels of CD11b in absence of CXCL-8 and *E. coli*, neutrophils (2×10^5^ cells/well) were pretreated with LT (0, 20 and 500 ng/mL) for 2 h. After pretreatment, neutrophils were incubated with CXCL-8 (1 μg/mL) or pHrodo^TM^ red *E. coli* (6×10^6^ particles) at 37°C for another 2 h. Then, neutrophils were washed 3 times with cold PBS to remove excess CXCL-8 or *E. coli* and stained with FITC-conjugated anti-porcine CD11a antibody (1:10 dilution, Bio-Rad) or FITC-conjugated anti-porcine CD11b (1:10 dilution, Abcam) antibody in PBS + 1%FCS for 40 min on ice. Upon washing to remove any unbound antibodies, the neutrophils were stained with 1 μM Sytox^TM^ Blue and measured using flow cytometry (Cytoflex, Beckman Coulter). The data were analyzed using CytExpert software (Beckman Coulter).

qPCR

Neutrophils were treated with different concentrations of LT for 2 or 8 h. Next, neutrophils were collected and total RNA was isolated using the Qia Shredder and RNeasy Mini Kit (Qiagen) according to the manufacturer’s guidelines. The RNA concentration and purity were determined by microvolume UV-Vis spectrophotometry (DeNovix, Wilmington, DE, USA) and the RNA integrity was evaluated by agarose gel electrophoresis. RNA (500 ng) was treated with RQ1 RNase-Free DNase (Promega) and subsequently reverse transcribed into cDNA using SuperScript III Reverse Transcriptase kit (Invitrogen) in the presence of a recombinant ribonuclease inhibitor (RNase OUT; Invitrogen) according to the manufacturer’s instructions. The resulting cDNA served as a template for the qPCR assay. Primers (table S1) were designed with Primer-BLAST (NIH, USA) or taken from literature and synthesized by Integrated DNA Technologies (IDT, Coralville, IA). Quantitative PCR was performed using 25 ng cDNA template at 60°C annealing temperature using a StepOnePlus real-time PCR system (Applied Biosystems) with SYBR green master mix (Applied Biosystems), following the protocol provided by the manufacturer. The final volume of qPCR mix is 20 μL. The cycle conditions were 1 cycle of 10 min at 95°C and 40 cycles of 15 s at 95°C, 30 s at the annealing temperature (60°C), and 30 s at 72°C. After the cycles, the melt curve analysis was performed. The results were analyzed by the double delta threshold cycle method and normalized to the expression level of the reference genes (β-actin and GAPDH) and to the control condition. Reference genes were selected based on geNorm analysis using qBase+ software.

### Cytokine and chemokine secretion

Neutrophils (2 × 10^6^ cells) were treated with different concentrations of LT for 8 h, upon which the supernatant was collected and centrifuged at 400 g for 5 min. The IL-6, CXCL-8 and TNF-α concentration in the cell-free supernatant were measured using commercial DuoSet ELISA kits (R&D systems) according to the manufacturer’s instructions. For the role of PKA and ERK1/2 signaling in LT-induced cytokine secretion, neutrophils were preincubated with diluent control (DMSO), 10 μM H89 or U0126 for 2 h at 37°C before LT was added to the neutrophils.

### Quantifying NETs formation

Neutrophils were seeded in 96-well plates (2×10^5^ cells/well), incubated at 37°C with 5% CO_2_ for 1 h and then stimulated with various concentrations of LT (0, 4, 20, 100, and 500 ng/mL). Cells were imaged using a Thunder microscope upon 3, 6, and 9 h of incubation.

NETs formation was quantified by detecting DNA release from the neutrophils. Neutrophils (2×10^5^ cells/well) were incubated at 37°C with 5% CO_2_ for 1 h and then treated with 0, 20, and 500 ng/mL LT for an additional 8 h. PMA (5 μg/mL) was included as a positive control. For the inhibition assay, neutrophils were preincubated with 10 μM H89 or U0126 for 2 h at 37°C before LT was added to neutrophils. The neutrophils were then stained with Sytox Green (1 μM, Invitrogen) for 15 min at 37°C, followed by a Syto Red stain (1 μM, Invitrogen) for another 15 min at 37°C. Images were acquired using a Thunder microscope (Leica) and analyzed using Image J software to quantify NETs formation.

### Confocal microscopy to examine NETs formation

Neutrophils were seeded (2×10^6^ cells/well) on poly-L-lysine-coated coverslips and allowed to rest for 1 h at 37°C. Subsequently, the neutrophils were treated with varying concentrations of LT (0, 20, and 500 ng/mL) or PMA (5 μg/mL) for an additional 8 h. Following treatment, the neutrophils were fixed with 4% paraformaldehyde in PBS for 10 min at 37°C and then permeabilized with 0.1% Triton X-100 in PBS for 20 min. Upon three washes with PBS and blocking with 1% BSA containing 5% goat serum in PBS for 2 h at 37°C, the cells were stained overnight at 4°C with rabbit anti-MPO antibodies (diluted 1:200, R&D systems). Upon washing to remove unbound antibodies, the neutrophils were stained with FITC-conjugated goat anti-rabbit IgG (diluted 1:200, Sigma) antibodies for 2 h at 37°C. Upon washing, nuclei were counterstained with Hoechst 33342 for 5 min at room temperature. Finally, the neutrophils were washed and mounted to observe MPO using confocal microscopy (Leica). The acquired images were analyzed using Image J software.

### Antimicrobial activity of NETs

The antimicrobial activity of NETs was measured following the method described in a previous study with some modifications.^71^ Neutrophils were seeded at 2 × 10^5^ cells/well in 96-well plates and incubated at 37°C and 5% CO_2_ for 1 h. Then, neutrophils were treated with 0 or 500 ng/mL LT for another 8 h. PMA (5 μg/mL) was used as a positive control. As a negative control, neutrophils were preincubated with the phagocytosis inhibitor cytochalasin D (10 µg/ml, Sigma Aldrich, Taufkirchen) for 20 min. The neutrophil supernatant was then removed upon centrifugation (400 g, 5 min) and 100 μL RPMI 1640 containing the porcine ETEC strain GIS26 (F4^+^, LT^+^STa^+^STb^+^)^23^ or the *E. coli* lab strain HB101 at 2 × 10^6^ CFU/mL was added to each well and incubated at 37°C for 120 min. Growth of the bacteria was monitored by measuring the OD_600_ at 20 min intervals (Tecan Spark).

### Western blotting

To analyze phosphorylation of PKA, neutrophils (2×10^6^ cells) were treated with LT (0, 20 or 500 ng/mL) for 2 or 8 h. Then, the cells were lysed using 60 μL RIPA buffer containing a protease and phosphatase inhibitor cocktail (1:100; Thermo Fisher Scientific) and EDTA (0.05 M; Thermo Fisher Scientific). Upon determining the protein concentration using a BCA kit (Thermo Fisher Scientific), proteins were denatured by adding 10 μL 6× loading buffer, 3 μL β-mercaptoethanol and heating for 5 min at 95°C. Then, equal amounts of proteins were separated on a 10% SDS – PAGE and subsequently transferred onto PVDF membranes (Amersham). After blocking in PBS + 0.05% Tween 20 + 5% BSA for 1 h at room temperature, membranes were probed overnight at 4°C with rabbit polyclonal anti-PKA C-α (1:1000, Cell signaling Technology), rabbit polyclonal anti-phospho- PKA C (Thr197, 1:500, Cell signaling Technology), rabbit monoclonal anti-phospho-PKA substrate (1:500, Cell signaling Technology), rabbit monoclonal anti-phospho-PKC (gamma Thr514, 1:500, Cell signaling Technology), rabbit polyclonal anti-p44/42 MAPK (1:1000, Cell signaling Technology), rabbit polyclonal anti-phospho-p44/42 MAPK (Thr202/Tyr204, 1:300, Cell signaling Technology) or rabbit monoclonal anti-β-actin (1:1000; Cell Signaling Technology) antibodies. Then, membranes were washed 3 times and incubated with HRP-conjugated porcine anti-rabbit IgG antibody (1:1000, Dako) for 1 h at room temperature. After washing 3 times, protein bands were visualized using a SuperSignal^TM^ ultimate sensitivity chemiluminescent substrate kit (Thermo Fisher Scientific) according to the manufacturer’s instructions. The relative band intensities were determined using Image Lab software (Bio-Rad). To evaluate the role of certain signaling pathways, the neutrophils were preincubated with diluent control 10 μM H89, Akt inhibitor XI (Santa Cruz Biotechnology), Gö 6983 (MedChemExpress) or U0126 for 2 h at 37°C before LT was added.

### Statistical analysis

Statistical analysis was performed with IBM SPSS Statistic 26 (USA). Homogeneity of variances was assessed with Levene’s test. Student’s T test, Friedman test or one-way ANOVA with posthoc Tukey test were used for statistical analysis as indicated in the figure legends. Data are presented as the mean ± standard deviation (SD). A p-value <0.05 was considered significant.

## Supplementary Material

Supplemental Material

## Data Availability

The data that support the findings of this study are available from the corresponding author upon reasonable request. Source data are provided with this paper.
