# Supplementary material for: Enterotoxigenic Escherichia coli heat labile enterotoxin affects neutrophil effector functions via cAMP/PKA/ERK signaling
Source: Gut Microbes. 2024 Sep 16;16(1):2399215. doi: 10.1080/19490976.2024.2399215 (PMC11407407; doi:10.1080/19490976.2024.2399215)
Supplement: Supplemental Material [file KGMI_A_2399215_SM1021.zip › Clean version_Supplementary Materials.docx]

Supplementary Material

##
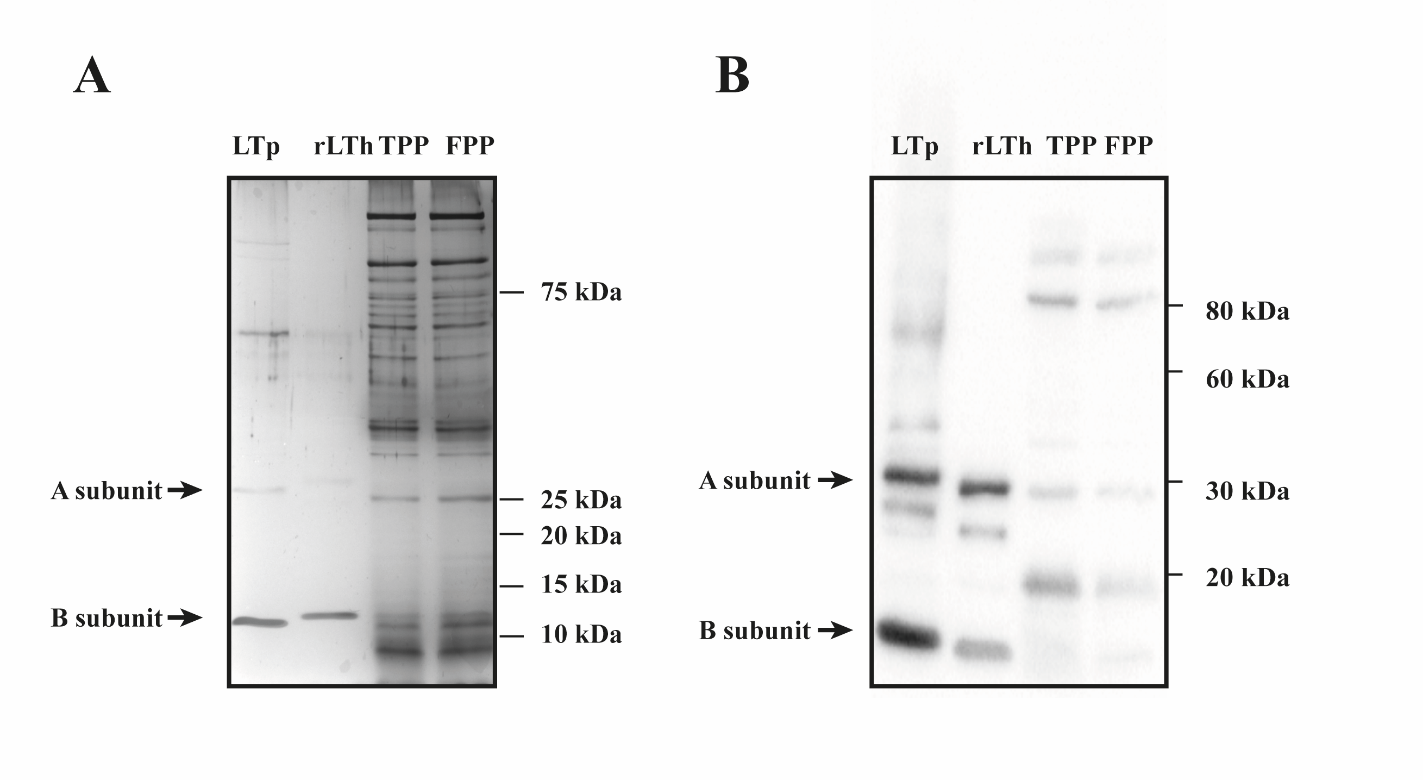
Supplementary Figures

**Supplementary Figure 1.** Expression and characterization of the heat labile enterotoxin LT. (A) silver-stained SDS-PAGE gel. Lane 1: LT (2 μg) purified from culture supernatant of the porcine wild type ETEC strain IMM07; lane 2: recombinant LTh (2 μg); lane 3: Total precipitated proteins in 55% ammonia sulfate solution (TPP); lane 4: Precipitated proteins flowing through the galactose column (FPP). (B) Immunoblot of the A and B subunit of heat labile enterotoxin. Lane 1, purified LT; lane 2, recombinant LTh; lane 3, input; lane 4, flow through. LTp = LT from a porcine ETEC strain, LTh = LT from a human ETEC strain.


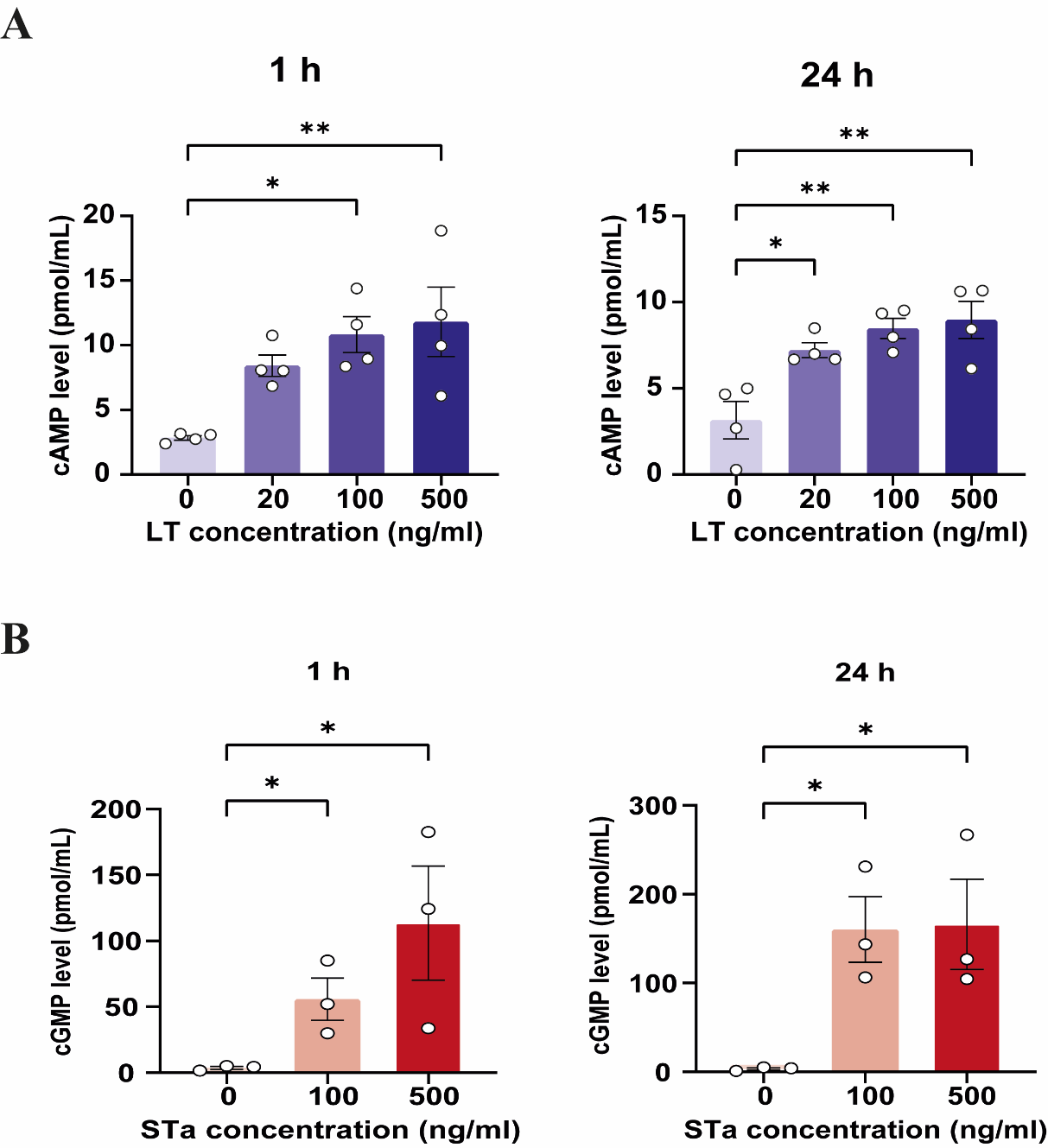


**Supplementary figure 2.** The bioactivity of purified LT and synthesized STa. (A) The intracellular cAMP levels in IPEC-J2 cells after treatment with purified LT at the indicated concentrations for 1 or 24 h. (B) The intracellular cGMP levels in T84 cells after treatment with synthesized STa for 1 or 24 h. At least 3 independent experiments were performed. The bars represent the mean ± SD. The data were analyzed with One-way ANOVA with a Tukey test. **p* < 0.05, to the control condition.


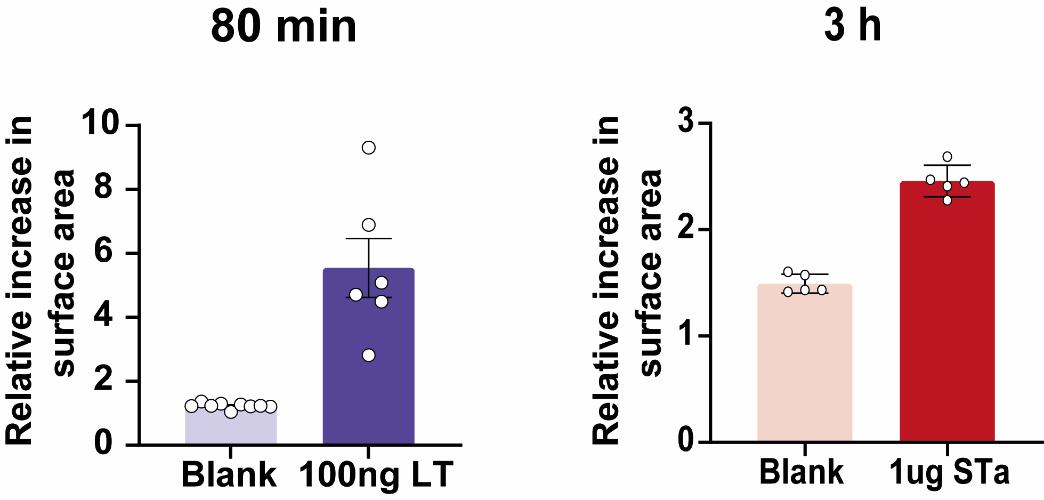


**Supplementary figure 3.** LT and STa induced swelling of porcine jejunal enteroids. Quantitative analysis of the relative change in surface area at the indicated timepoint as compared to the initial timepoint. The bars represent the mean ± SD.

The representative videos (Movie S1-S3) of the swelling assay have been uploaded online and can be downloaded using the link below:

https://drive.google.com/drive/folders/1-qDA9m5iqzfur1Ibs19L8DHVTvtUovl_?usp=sharing


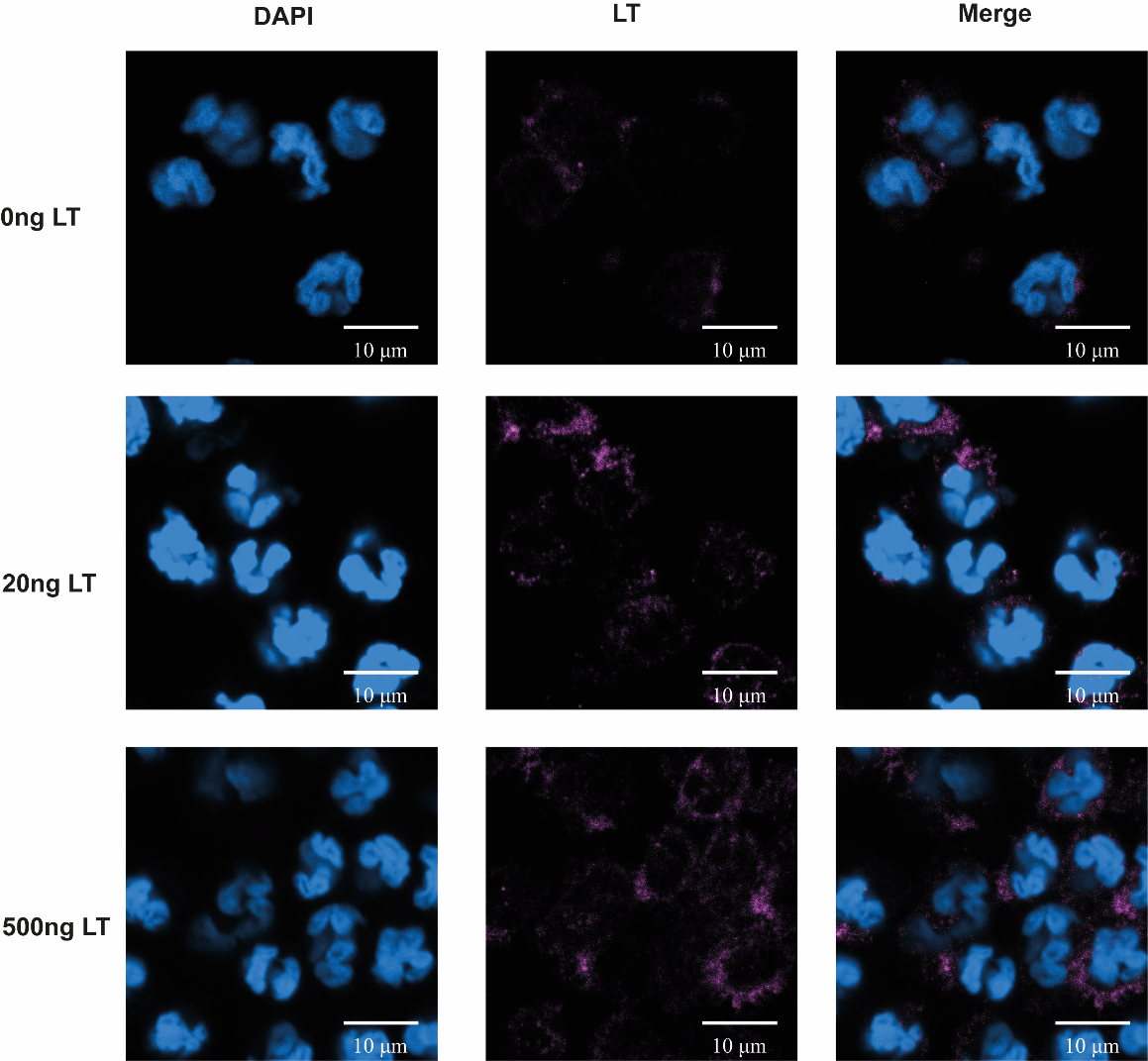


**Supplementary figure 4.**  Confocal microscopy images of LT localization on neutrophils after treatment with LT for 2 h.


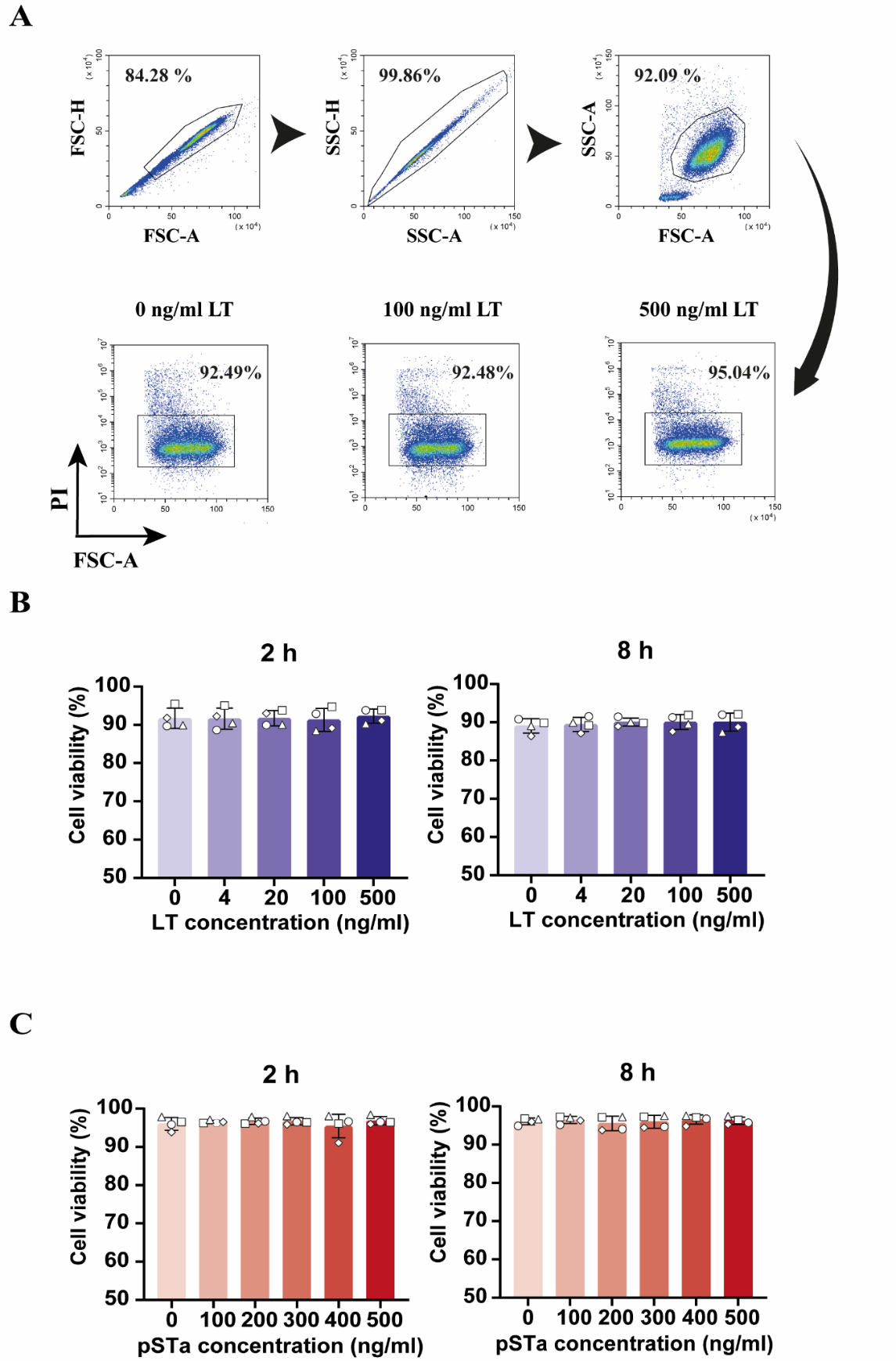


**Supplementary figure 5.**  LT and pSTa do not trigger cell death in neutrophils. (A) The gating strategy used to select neutrophils by flow cytometry and representative dot plots of neutrophil viability upon treatment with LT or STa for 8 h. (B-C). Neutrophils (2x10^5^) were incubated with LT (B) or pSTa (C) at the indicated concentration for 2 or 8 h at 37°C. The cell viability of neutrophils was analysed by propidium iodide (PI) staining and flow cytometry. n = 4 individual blood donors. The bars represent the mean ± SD. The data of were analysed with one-way ANOVA with a posthoc Tukey test.


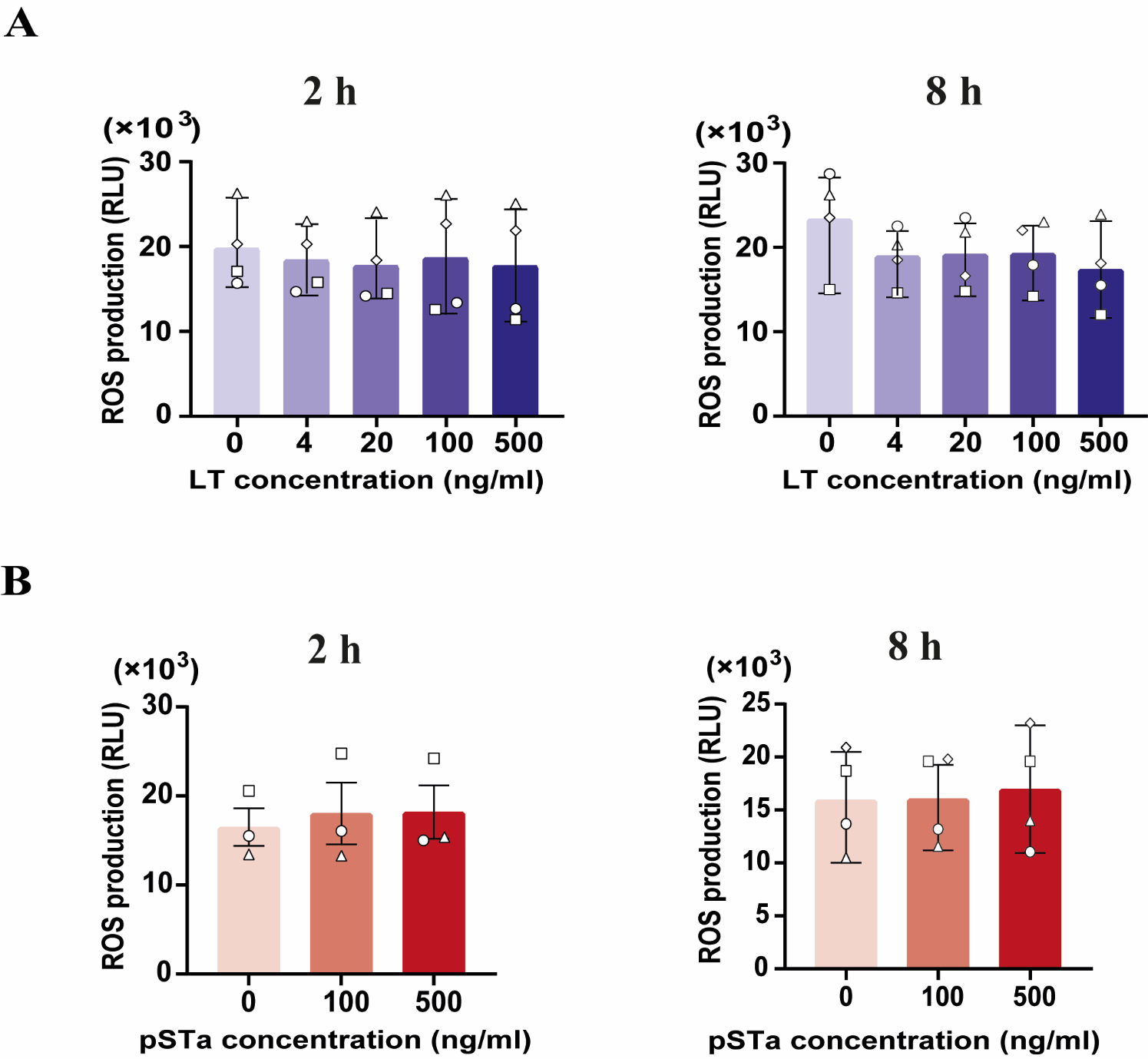


**Supplementary figure 6.**  LT and pSTa do not affect the ROS production induced by PMA. (A) Neutrophils (2x10^5^) were pre-treated with 0-500 ng/mL LT or (B) 0-500 ng/mL pSTa for 2 or 8 h at 37 °C, and then incubated with 50 μM PMA for another 2 h. The ROS production was measured in a chemiluminescence assay. RLU: relative light units. n = 3 to 4 individual blood donors. The bars represent the mean ± SD. Data were analyzed with one-way ANOVA with a post hoc Tukey test to compare LT or pSTa treatment groups to the control group.


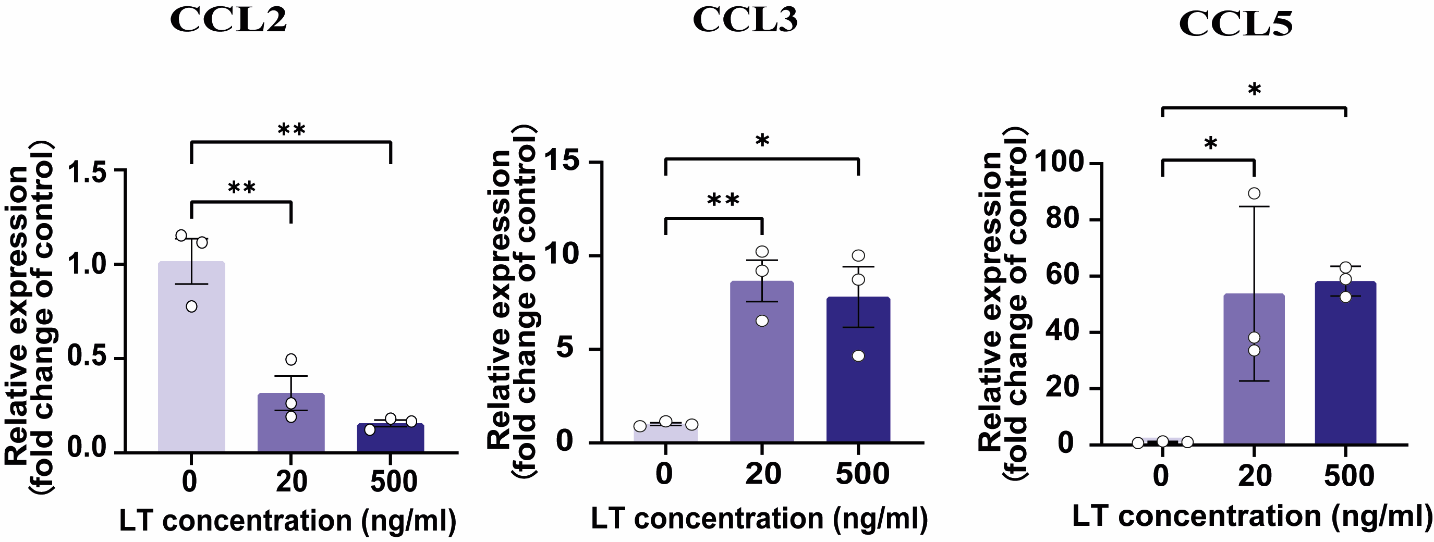


**Supplementary figure 7.** Neutrophils respond to LT by increased production of chemokines. Neutrophils (2x10^6^) were treated with LT at the indicated concentrations for 2 h. Changes in CCL2, CCL3 and CCL5 transcript levels were evaluated by qPCR. n = 3 individual blood donors. The bars represent the mean ± SD. Date were analyzed with one-way ANOVA and a posthoc Tukey test. **p* < 0.05, ***p* < 0.01for comparisons between cells treated with LT and medium alone.


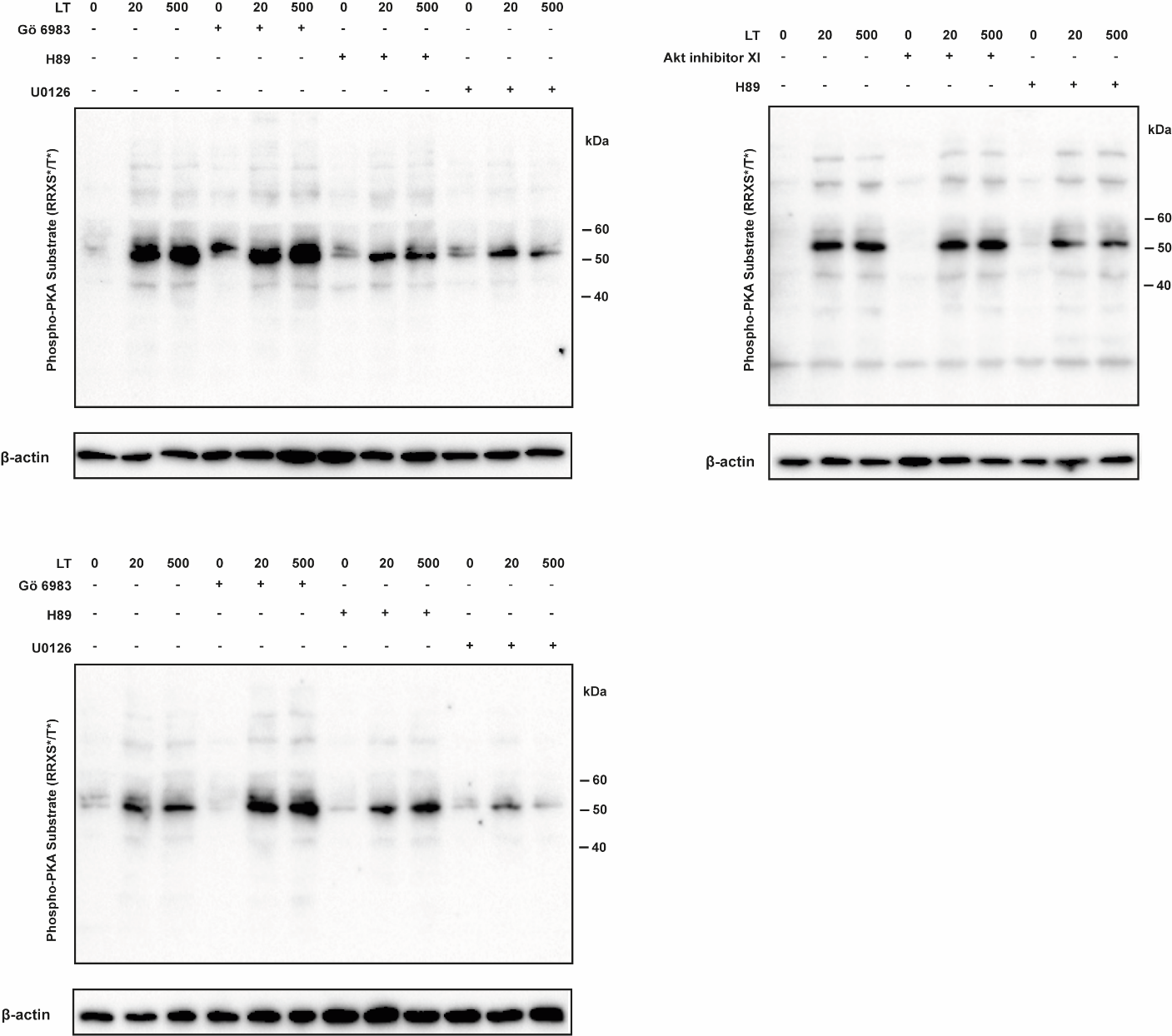


**Supplementary figure 8.**  LT-induced activation of the cAMP/PKA signalling pathway in porcine neutrophils. Representative immunoblots of phospho-PKA substrates in neutrophils (2x10^6^) pre-treated with 10 μM H89, 10 μM U0126, 10 μM Akt inhibitor XI or Go6983 for 2 h, and then treated with LT for 2 h at the indicated concentrations.


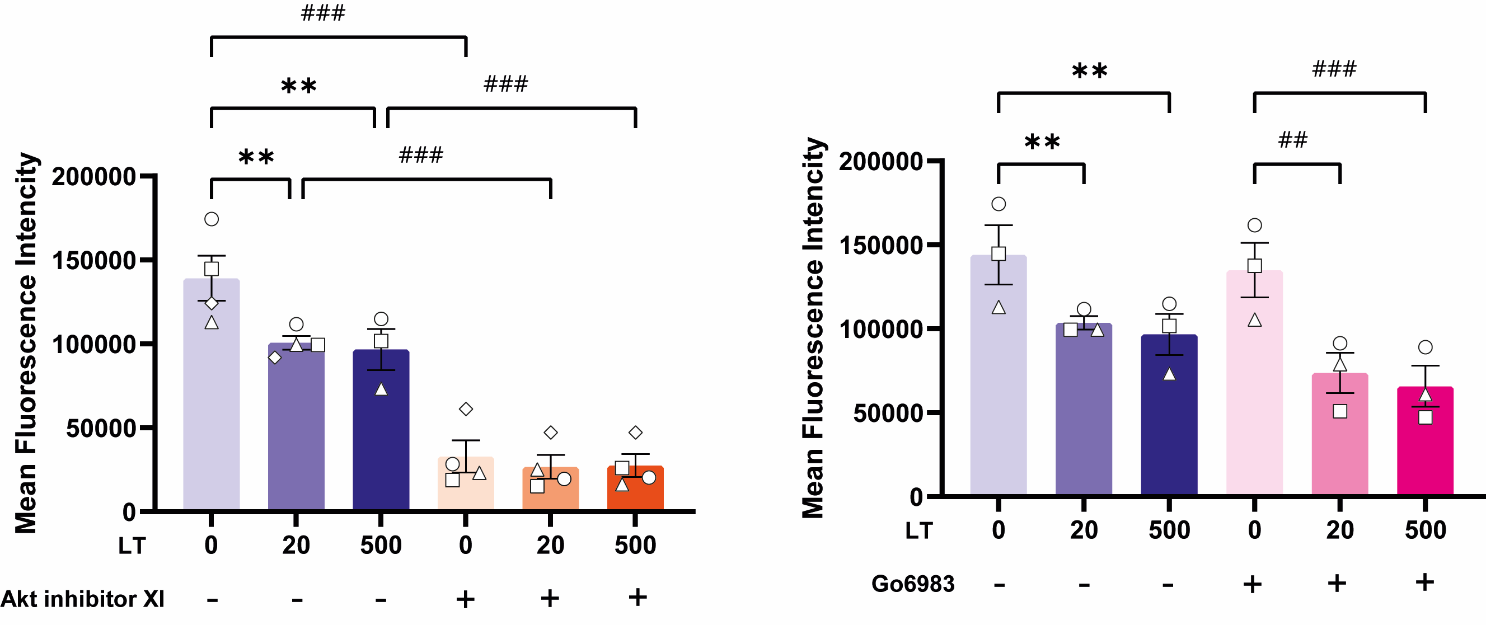


**Supplementary figure 9.** Akt and PKC are not involved in the LT-induced reduction of phagocytosis by neutrophils. At least 3 independent experiments were performed, and the neutrophils for each experiment were isolated from different pigs. Each value was expressed as mean ± SD. One-way ANOVA with a Tukey test was used to compare LT or STa treatment groups to no toxin control group. **p* < 0.05, ***p* < 0.01, ****p* < 0.001. A paired T test was used to compare two groups with or without Akt inhibitor XI or Go6983. #*p* < 0.05, ##*p* < 0.01, ###*p* < 0.001.

Table S1. Sequences and concentrations of primers used in the qPCR assay.

| Target | Accession number | Primer Sequence | Primer concentration | Reference |
| --- | --- | --- | --- | --- |
| β-actin | AY550069 | **Fw:** TCATCACCATCGGCAACG | 250 nM | [1] |
|  |  | **Rv:** TTCCTGATGTCCACGTCGC | 250 nM |  |
| GAPDH | AF017079 | **Fw:** GGGCATGAACCATGAGAAGT | 250 nM | [2] |
|  |  | **Rv:** AAGCAGGGATGATGTTCTGG | 250 nM |  |
| IL1β | NM_214055.1 | **Fw:** AGCCCAATTCAGGGACCCTAC | 250 nM | - |
|  |  | **Rv:** TGCCTGATGCTCTTGTTCCA | 250 nM |  |
| IL6 | NM_214399.1 | **Fw:** CCTGAGATTGATGCCGTCCA | 250 nM | - |
|  |  | **Rv:** TCTTCAAGCCGTGTAGCCAT | 250 nM |  |
| TNFα | NM_214022 | **Fw:** ACTGCACTTCGAGGTTATCGG | 250 nM | [3] |
|  |  | **Rv:** GGCGACGGGCTTATCTGA | 250 nM |  |
| CXCL8 | NM_213867.1 | **Fw:** GACCCCAAGGAAAAGTGGGT | 250 nM | - |
|  |  | **Rv:** TGACCAGCACAGGAATGAGG | 250 nM |  |
| CCL2 | NM_214214.1 | **Fw:** CCAGGACTCCATAAGCCACC | 250 nM | - |
|  |  | **Rv:** CAATGTGCCCAAGTCTCCGT | 250 nM |  |
| CCL3L1 | NM_001009579.1 | **Fw:** CCTCGCAAATTCGTAGCCGA | 250 nM | - |
|  |  | **Rv:** TCAGCTCCAGGTCAGAGATGT | 250 nM |  |
| CCL5 | NM_001129946.1 | **Fw:** TGCTTCTTGCTCTTGTCCCA | 250 nM | - |
|  |  | **Rv:** GTGCCAAGGGTCCAAAGTTC | 250 nM |  |

Fw, Forward primer; Rv, reverse primer.

**Supplemental references**

[1] K. Von der Hardt, M.A. Kandler, L. Fink, E. Schoof, J. Dotsch, O. Brandenstein, R.M. Bohle, and W. Rascher, High frequency oscillatory ventilation suppresses inflammatory response in lung tissue and microdissected alveolar macrophages in surfactant depleted piglets. Pediatr Res 55 (2004) 339-346.

[2] V. Melkebeek, F. Verdonck, B.M. Goddeeris, and E. Cox, Comparison of immune responses in parenteral FaeG DNA primed pigs boosted orally with F4 protein or reimmunized with the DNA vaccine. Vet Immunol Immunop 116 (2007) 199-214.

[3] L. Hermans, S. De Pelsmaeker, S. Denaeghel, E. Cox, H.W. Favoreel, and B. Devriendt, beta-Glucan-Induced IL-10 Secretion by Monocytes Triggers Porcine NK Cell Cytotoxicity. Front Immunol 12 (2021).
